# Supplementary material for: Dispersal in heterogeneous environments drives population dynamics and control of tsetse flies
Source: Proc Biol Sci. 2021 Feb 3;288(1944):20202810. doi: 10.1098/rspb.2020.2810 (PMC7893214; doi:10.1098/rspb.2020.2810)
Supplement: Data and model details [file rspb20202810supp1.docx]

**Dispersal in heterogeneous environments drives population dynamics and control of tsetse flies**

H. Cecilia, S. Arnoux, S. Picault, A. Dicko, M.T. Seck, B. Sall, M. Bassène, M. Vreysen, S. Pagabeleguem, A. Bancé, J. Bouyer, P. Ezanno

Proc. Roy. Soc. B: 10.1098/rspb.xxxx.xxxx

**Supporting Information**

**Index table**

[1. Life cycle and study area 2](#_Toc52977736)

[Fig. S1. Local and general tsetse fly population dynamics applied to the Niayes in Senegal 2](#_Toc52977737)

[2. Experimental data 2](#_Toc52977738)

[**2.1 Mortality, fecundity, and length of the pupal period** 2](#_Toc52977739)

[Table S1. Pupa emergence data 3](#_Toc52977740)

[Fig. S2. Data and predictions for temperature-dependent processes 3](#_Toc52977741)

[Fig. S3. Calibration of male mortality rate 4](#_Toc52977742)

[**2.2 Dispersal** 4](#_Toc52977743)

[Fig. S4. Distance between releases and captures of marked tsetse flies 5](#_Toc52977744)

[**2.3 Population age structure** 5](#_Toc52977745)

[3. Additional information on input data generation 5](#_Toc52977746)

[**3.1 Carrying capacities** 5](#_Toc52977747)

[**3.2 Temperature** 6](#_Toc52977748)

[4. Modelling tsetse fly population dynamics 6](#_Toc52977749)

[**4.1. Within-cell dynamics** 6](#_Toc52977750)

[**4.2. Spatial dispersal** 7](#_Toc52977751)

[Fig. S5. Dispersal rate 8](#_Toc52977752)

[**4.3 Model setting** 8](#_Toc52977753)

[Table S2. Parameter values 8](#_Toc52977754)

[**4.4 Additional results on the scenario without control** 9](#_Toc52977755)

[Fig. S6. Model predictions for the scenario without control 9](#_Toc52977756)

[Fig. S7. Variability of tsetse fly population size 10](#_Toc52977757)

[5. Sensitivity analysis 10](#_Toc52977758)

[Table S3. Definition of aggregated outputs 10](#_Toc52977759)

[Fig. S8. Scaling down of temperature variations for the global sensitivity analysis 11](#_Toc52977760)

[Fig. S9. Sensitivity of the population size to variations in temperature and carrying capacity 11](#_Toc52977761)

[Fig. S10. Age structure showed almost no variation with parameter variations 12](#_Toc52977762)

[Fig. S11. Global sensitivity analysis 14](#_Toc52977763)

[6. Additional information about control strategies 14](#_Toc52977764)

[Fig. S12. Local efficacy of a homogeneous control 15](#_Toc52977766)

[Fig. S13. Local efficacy of a heterogeneous control 15](#_Toc52977767)

[7. Additional results when neglecting fly spatial dispersal 15](#_Toc52977768)

[Fig. S14. To be compared to Fig. 2. 16](#_Toc52977769)

[Fig. S15. To be compared to Fig. S12 16](#_Toc52977770)

[Fig. S16. To be compared to Fig. S13 17](#_Toc52977771)

[Fig. S17. To be compared to Fig. 3 17](#_Toc52977772)

[Fig. S18. To be compared to Fig. 4 18](#_Toc52977773)

[8. Code sources and data files 18](#_Toc52977774)

# 1. Life cycle and study area

**
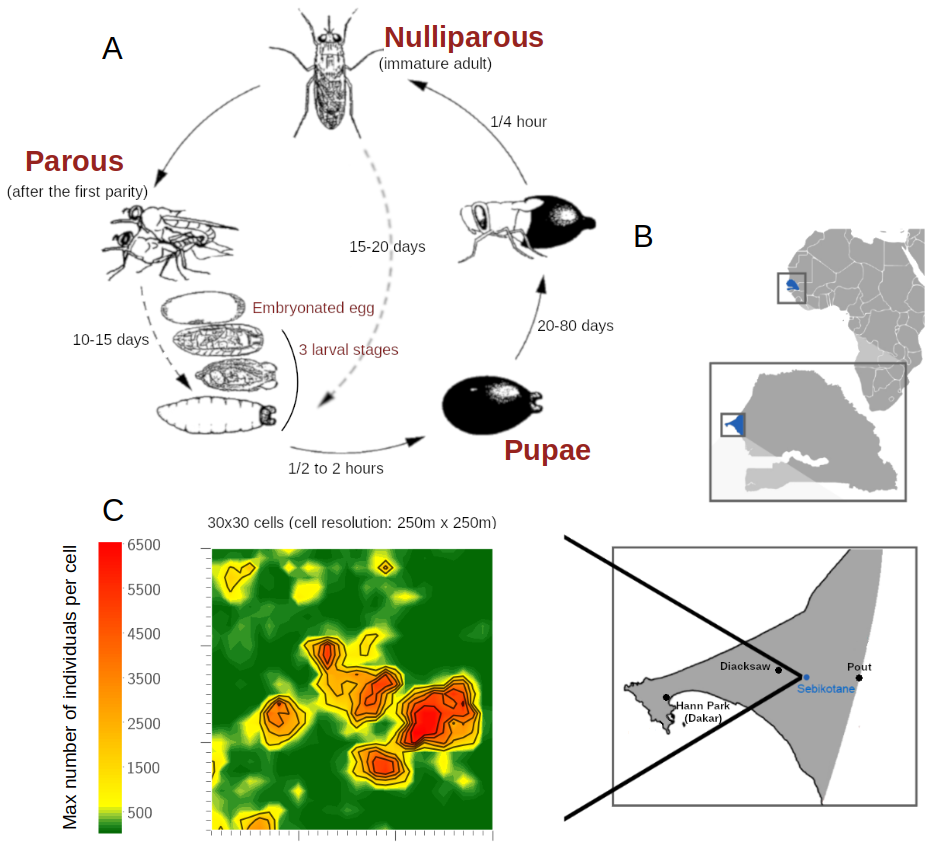
**

### **Fig. S1.** Local and general tsetse fly population dynamics applied to the Niayes in Senegal. (A) life cycle of tsetse flies as it occurs locally within each cell (drawn by D. Cuisance). (B) Map of Senegal with locations where field data were collected. (C) Simulated area, highlighting the spatial heterogeneity resulting in different local carrying capacities *k_c_*.

# 2. Experimental data

## **2.1 Mortality, fecundity, and length of the pupal period**

To assess the effect of temperature on the life cycle of *G. p. gambiensis,* we used data on adult mortality, first larviposition period (time between emergence and first larviposition day), and subsequent inter-larval periods (time between reproductive cycles). These data were initially produced in order to assess the maximum critical temperature for the flies, and the colony was maintained at 24°C. Therefore, most data used to estimate female mortality were obtained at 24°C and none at lower temperatures (Fig. S2A). We considered a constant mortality before 24°C and fitted a weighted log-linear model to observed mortality rates. These mortality rates were obtained by applying a Kaplan-Meier estimator to laboratory survival data.

In addition, the effect of temperature on the length of the pupal period was measured under experimental conditions at the Centre International de Recherche-Développement sur l’Elevage en zones Subhumides (CIRDES) in Bobo-Dioulasso, Burkina Faso, in 2009 (Table S1). Pupae were hold in climate controlled rooms until emergence. For temperatures below and above 25°C, the pupae underwent two phases: the first 20 days around 25°C (phase 1), and the subsequent days until emergence at a different temperature (phase 2). For each temperature-varying group, a control group was held at constant temperature around 25°C until emergence.

New equations were calibrated for temperature-dependent processes of the life cycle of *G. p. gambiensis* combining published and new observed data (Eq. S7, Fig. S2-S3).

### **Table S1.** Pupa emergence data, used for calibration of the development rate (males and females are undifferentiated at this stage in the model).

|  | Control group (constant temperature) | | | | Temperature-varying group | | | |
| --- | --- | --- | --- | --- | --- | --- | --- | --- |
| Sex | Temperature (°C) | Time to hatch (days) | Number of flies | Daily development rate* (day^-1^).10^-2^ | Temperature phase 2 (after 20 days, °C) | Range of time spent in phase 2 before hatching (days) | Number of flies | Estimated pupal duration if constant temperature*† (days) |
| F | 25.40 +- 1.08 | 25 | 6 | 3.83 | 15.0 +- 0.7 | [17-28] | 186 | 98.31 |
|  |  | 26 | 30 |  |  |  |  |  |
|  |  | 27 | 12 |  |  |  |  |  |
|  | 25.41 +- 1.18 | 28 | 16 | 3.48 | 20.11 +6 0.32 | [15-23] | 178 | 62.53 |
|  |  | 29 | 30 |  |  |  |  |  |
|  |  | 30 | 2 |  |  |  |  |  |
|  | 25.80 +- 0.80 | 26 | 33 | 3.78 | 27.5 +- 0.8 | [5-6] | 188 | 23.40 |
|  |  | 27 | 28 |  |  |  |  |  |
| M | 25.40 +- 1.08 | 27 | 9 | 3.55 | 15.0 +- 0.7 | [25-29] | 180 | 95.04 |
|  |  | 28 | 35 |  |  |  |  |  |
|  |  | 29 | 22 |  |  |  |  |  |
|  | 25.41 +- 1.18 | 30 | 23 | 3.26 | 20.11 +6 0.32 | [21-25] | 165 | 64.80 |
|  |  | 31 | 40 |  |  |  |  |  |
|  |  | 32 | 3 |  |  |  |  |  |
|  | 25.80 +- 0.80 | 28 | 23 | 3.50 | 27.5 +- 0.8 | [6-8] | 155 | 23.40 |
|  |  | 29 | 35 |  |  |  |  |  |

*Weighted mean: full data is provided for control groups. Only duration ranges are provided for temperature-varying groups for brevity. There were 3 replicates for each temperature-varying group. The development duration at a constant temperature is duration_Tconst_ = 1 / dev.rate_Tconst_. Thus, the daily development rate for the control group is dev.rate_control_ = 1 / duration_control_. We deduced the pupal duration if temperature of phase 2 had been applied over the whole development.

†To estimate the total pupal duration if temperature had been constant, we applied the average development rate of the control group to the first 20 days. After 20 days, the proportion of the development still to be done is duration_Phase2_*dev.rate_Tconst_. It corresponds to (1 – 20*dev.rate_control_). It follows that duration_Tconst_ = duration_Phase2_ / (1 – 20 * dev.rate_control_).


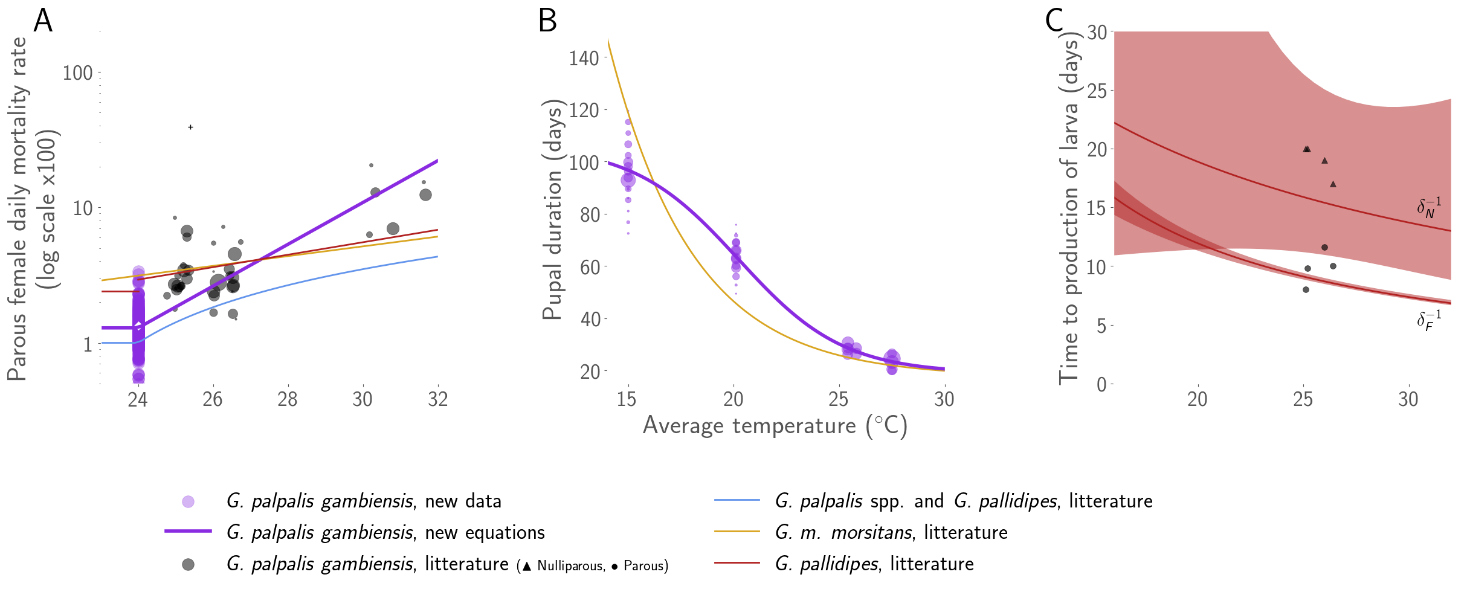


### **Fig. S2.** Data (symbols) and predictions (lines) fitted to the new data (if relevant) and from literature for temperature-dependent processes of the model. A: parous female daily mortality rate (in log_10_-scale); B: pupal duration (in days) with duration = 1 / development rate (Eq. S7, Table S2); C: time to larviposition for nulliparous (N, upper curve, triangles) and parous females (F, lower curve, dots). Data from Pagabeleguem et al. (2016) is shown in grey (the cross in A was considered an outlier). New data on *G. p. gambiensis* (from FAO/IPCL and CIRDES) is shown in purple, with the barycentre of mortality rate at 24°C highlighted as a white-filled diamond. Purple thick lines are the newly calibrated equations used in the population dynamics model. In A, both grey and purple data were used to fit the new equation. Equation from Barclay and Vreysen (2011, 2013) is shown in blue. Hargrove’s equations (2004) obtained for different species are shown in light and dark orange. In B, the Hargrove’s equation has been fit based on data from Phelps and Burrow’s (1969). Filled areas in C correspond to prediction intervals of Hargrove’s equation. The few new data available for the time to larviposition were consistent with Hargrove’s equation, which thus was used.


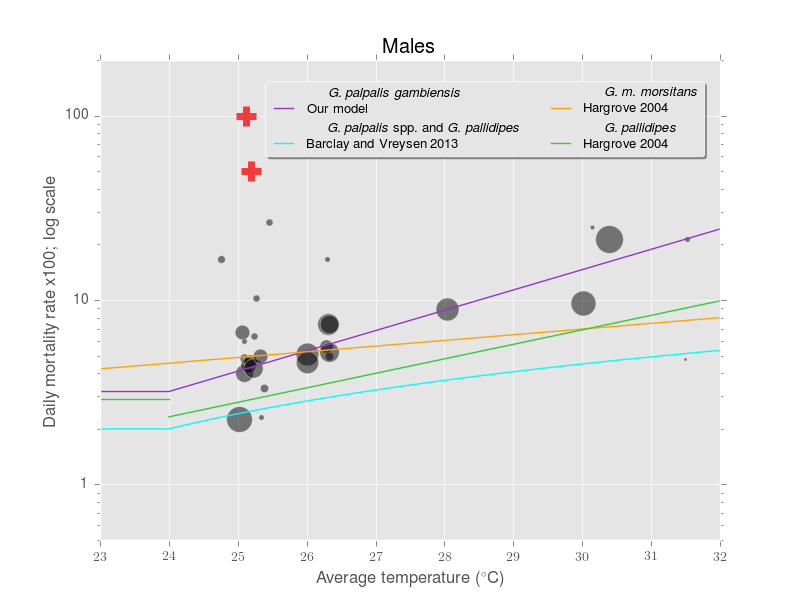


### **Fig. S3.** Calibration of male mortality rate. Data and comparison to literature. Red cross: outlier. Point size proportional to sample size.

## **2.2 Dispersal**

To assess the dispersal of *G. p. gambiensis*, we used release-recapture data of marked (different colours) sterile males from October 2010 to December 2012 (Pagabeleguem 2012, Pagabeleguem et al. 2015). Mass-reared male flies from the CIRDES colony were shipped as irradiated and chilled pupae to Senegal. After emergence, they were released twice a month and monitored in four areas: Parc de Hann in Dakar, Diacksaw Peul, Pout, and Kayar (Fig. S1B, Fig. S4). Two release points were selected per location (in suitable vs. unsuitable habitats) and released flies were trapped using Vavoua traps (Laveissière & Grébaut 1990) that were deployed at intervals of 100-300 m up to 2 km from the release points. Traps were deployed before 9 am and collected after 4 pm 3 days later. The monitoring of a release stopped when less than 2 marked males were recaptured. We matched captures of marked males (disregarding wild individuals) with the most recent release of the same colour. We did not account for recaptures in the same trap as the release (because individuals that did not fly from the trap during the release were already discarded). We then divided the distance between the recapture and release traps by the time between the two events. Flies were regularly collected in the traps, which allowed a good estimation of the time of capture, instead of assigning it to the end of trap deployment. Out of 9863 recaptured flies, 2701 were assigned to a release this way. We discarded one outlier for which the daily distance was superior to 5.5km.


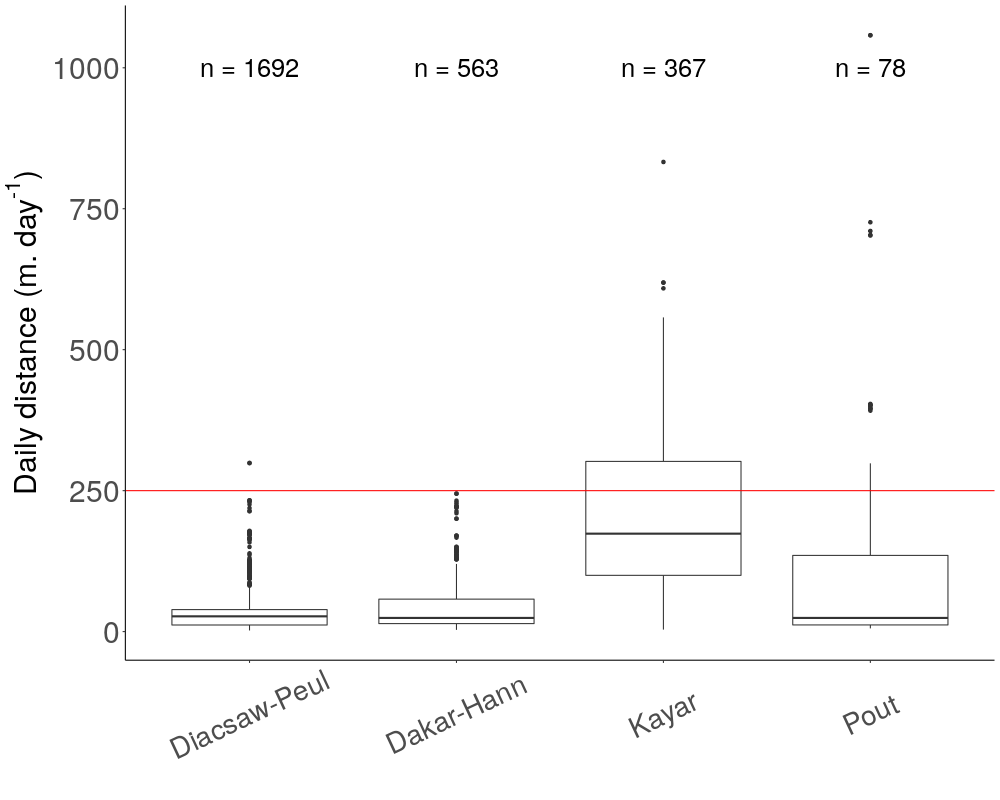


### **Fig. S4.** Distance (in meters) between releases and captures of marked tsetse flies, averaged by day for each of the observed locations (Niayes, Senegal).

## **2.3 Population age structure**

In another study, natural abortion rates were monitored in the Parc de Hann, Diacksaw Peul, Sebikotane, and Pout (Fig. S1B). In each site, 10 traps were deployed for three days every month from March 2008 to February 2009, and then every three months until September 2010 (Hann, Diacksaw) or December 2011 (Pout, Sebikotane). Flies were collected once to twice a day. All trapped live female flies that had not dried were dissected for ovarian ageing to determine their physiological age (n = 2,589; Challier, 1965). This female dataset (now provided as CSV files in the Sourcesup folder, see the link provided §8) was used to calculate the population age structure, to be compared to simulation results for partial validation of the model.

# 3. Additional information on input data generation

## **3.1 Carrying capacities**

Suitability Index (SI) - The first layer needed to estimate the carrying capacity is the habitat suitability index. We used this layer to determine the area where tsetse flies can survive (suitable habitats). A statistical analysis of the habitat was carried out using correlative species distribution models. The methodology used is based on the framework developed in the Niayes (Senegal) using the Maximum Entropy model (MaxEnt) (Dicko et al. 2014). MaxEnt is one of the most widely-used species distribution models. It is a machine learning method based on the information theory concept of maximum entropy (Elith et al. 2011). It fits a species distribution by contrasting the environmental condition where the species is present to the global environment characterized by some generated pseudo-absence data, also called the background. Occurrence data from already described entomological surveys were used as input. Characterization of the environment in the study area relied on the 5-year average, minimum, maximum, range, and standard deviation of four spatio-temporal layers (day land surface temperature (DLST), night land surface temperature (NLST), normalized difference vegetation index (NDVI), maximum middle infrared (MIR)), with the digital elevation model (DEM) added to the set of summarized variables. To account for the sampling bias present in the entomological data, a gaussian kernel based grid that gives more weight to more densely sampled areas was needed (Bouyer et al. 2009). Model complexity in the MaxEnt framework can be controlled using the beta regularization parameter. Five beta values (1, 1.5, 2, 3, 4) were tested. We performed multimodel inference using model averaging weighted by the Akaike Information Criterion (AIC) to choose the best model (Burnham & Anderson 2002; Warren & Seifert 2011). The final output was a suitability index that ranged between 0 (least suitable habitat) and 1 (most suitable habitat) defined for every patch, providing a quantitative indicator of the habitat preferences of *G. p. gambiensis* in the study area.

Apparent Density per Trap (ADT) - The second layer needed to estimate the carrying capacity is the apparent density of tsetse flies per trap per day (ADT), as measured using biconical traps considered here as substitution hosts (Dicko et al. 2015). ADT is considered as an apparent density because it does not depend only on the real fly density but also on fly dispersal, feeding frequency, and age structure which all impact trap catches. We predicted a 5-year average of tsetse ADT at a spatial resolution of 1km^2^ using a geostatistical model fitted against the computed suitability index. A negative binomial model with spatial random effects was used. Negative binomial models can be seen as an extension of the classical Poisson regression to account for over-dispersion in count data. In addition, because of the sampling bias and the clustering of observations in such entomological dataset, a spatial random effect using the Matern correlation structure was used (Cressie & Cassie 1993). Trap catch data from November 2007 to December 2010 were used, with a maximum of 77 flies trapped within a single trap in a day (2008-01-24). The same day, grouping traps located in the same square kilometre (10 traps in total), captures reached 223 flies.

Trapping efficiency - In the sites where ground releases were conducted, trap efficiency (measured as the probability that a trap catches a fly within 1 km² within 1 day (Barclay and Hargrove 2005)) was estimated at 0.03 (SD 0.04) and its variability in space and time was low.

## **3.2 Temperature**

We downloaded high spatial resolution (1km) daily air temperature data in the region from the Meteo1Km project (http://dailymeteo.org/content/about). This dataset results from the combination of MODIS Land Surface Temperature and meteorological datasets from around the world through the use of a spatio-temporal geostatistical model (Kilibarda et al. 2014). We then refined these grids to a 250m spatial resolution. However, these temperatures overestimate perceived temperatures and thus will result in erroneous model outcomes. Indeed, meteorological stations are located in areas mostly unsuitable for tsetse flies with higher temperatures than what they experience in their resting places where they stay most of the day. We thus used our own network of temperature data recorded every 15 minutes in some suitable patches, computed the average difference with satellite data for the same coordinates, and subtracted this value in all cells in order to correct the bias present in the initial data. We ended up with a more accurate model of air temperature in suitable areas that can be used to realistically simulate tsetse fly population dynamics.

# 4. Modelling tsetse fly population dynamics

## **4.1. Within-cell dynamics**

The mechanistic and deterministic compartmental model of tsetse spatio-temporal dynamics categorized individuals into stages: pupae (*P*, without differentiating males and females), nulliparous females (*N*), parous females with four ovarian ages (*F_1_*, *F_2_*, *F_3_*, *F_4+_*, Fig. 1, main text), and males (*M*).

The population size of life stage *S* at time *t* in cell *c* decreased with mortality, following a negative exponential model of instantaneous rate $\mu_{S,t,c}$ (Eq. S1, Table S2). Considering the lack of data on pupa mortality, we used a constant rate (Eq. S2, Table S2, Childs 2011). For adults, the log of mortality rates increased linearly with temperature ($\theta_{t,c}$ at time *t* in cell *c*) above 24°C (Hargrove 2004). Below this threshold, and for the range of temperatures observed in the field, the literature and the lack of data suggested a constant mortality rate (Eq. S3, Table S2). Age-dependence was featured by setting nulliparous mortality to twice that of parous females (Alderton et al. 2016, Eq. S4). Density-dependence occurred when the adult population exceeded the cell carrying capacity (Eq. S5-6, Table S2, Hargrove 2004).

$S_{t+\Delta t,c}=S_{t,c}exp\left( -\mu_{S,t,c}\Delta t \right)$ (Eq. S1)

with stage *S* ∈ {*P*, *N*, *F_x_*, *M*} and ovarian age *x* ∈ {1, 2, 3, 4+} (note that *µ_F,t,c_* applied irrespective of ovarian age), *∆t* = 1, and:

$\mu_{P}=m_{P}$ (Eq. S2)

$\mu_{X,t,c}=\left\{ \begin{aligned} \mu_{X,t,c}\left( \theta_{t,c}=24^{\circ}C \right),if\theta_{t,c}\leq24^{\circ}C \\ \mu_{X,t,c}\left( \theta_{t,c} \right),if\theta_{t,c}>24^{\circ}C \end{aligned} \right.$, *X* ∈ {*N, F*, *M*} (Eq. S3)

$\mu_{N,t,c}=2\mu_{F,t,c}$ (Eq. S4)

$\mu_{X,t,c}=\beta_{t,c}exp\left( m_{1,X}\theta_{t,c}+m_{2,X} \right)$, *X* ∈ {*F*, *M*} (Eq. S5)

$\beta_{t,c}=\left\{ \begin{aligned} 1,if\frac{A_{t,c}}{k_{c}}\leq1 \\ \frac{A_{t,c}}{k_{c}},if\frac{A_{t,c}}{k_{c}}>1 \end{aligned} \right.$, with $A_{t,c}=N_{t,c}+\sum_{i=1}^{4} F_{i,t,c}+M_{t,c}$ (Eq. S6)

In addition, individuals evolved within and between stages as a function of temperature, except adult males (*M*) who mate from 6-day post-emergence irrespective of temperature. Pupa development function $\delta_{P,t,c}$ was fitted to the data (Eq. S7, Table S2, Fig. S2B). For nulliparous and parous females, consistency of experimental data on the target species was checked against published equations (Hargrove 2004, Eq. S8, Table S2, Fig. 1, main text).

$\delta_{P,t,c}=\left( d_{1,P}+\frac{d_{2,P}-d_{1,P}}{1+exp\left( \frac{d_{3,P}-\theta_{t,c}}{d_{4,P}} \right)} \right)^{-1}$ (Eq. S7)

$\delta_{X,t,c}=d_{1,X}\left( \theta_{t,c}-24 \right)+d_{2,X}$, *X* ∈ {*N*, *F*} (Eq. S8)

Each stage was discretized into $n_{S}$ states, $n_{S}$ being the longest duration in stage *S* obtained with its development rate $\delta_{S}$ calculated at the minimum temperature of the year $min\left( \theta_{t,c} \right)$ (Fig. 1, main text). For higher temperatures, individuals made a leap forward in the development vector, the interval being determined by the integer part $l$ of *∆* (Eq. S9, Fig. 1, main text).

$\Delta_{S,t,c}=\delta_{S,t,c}\left( \theta_{t,c} \right)n_{S}$ (Eq. S9)

To avoid discretization artefacts, individuals were proportionally divided into two successive states according to the decimal part *q* of *∆* (Fig. 1, main text). Individuals who reached state $n_{S}$ (i.e. stage *S* is completed) evolved to the next stage. A pupa was produced at the end of both nulliparous and parous female stages. After the fourth ovarian age, parous females looped back to the start of *F_4+_* (i.e. stage *F_4+_* represented females who have produced at least 4 pupae).

## **4.2. Spatial dispersal**

The dispersal pattern was designed to favour suitable over hostile habitats to be conform with species behaviour. The proportion $p_{t,c}$ of flies leaving cell $c$ at time *t* was controlled by a sigmoidal density-dependent dispersal rate (Lloyd-Smith, 2010) (Eq. S10, Table S2):

$p_{t,c}=\left[ 1+exp\left( -g\left( \frac{A_{t,c}}{k_{c}}-1 \right) \right) \right]^{-1}$ (Eq. S10)

where *k_c_* denotes the carrying capacity in cell *c*, *A_t,c_* denotes the number of adults in cell *c* at time *t*, and *g* denotes a shape parameter set to 10 meaning that $p_{t,c}\left\{ \begin{aligned} \approx0,ifA_{t,c}<0.5k_{c} \\ \approx1,ifA_{t,c}>1.5k_{c} \\ 0.5,ifA_{t,c}=k_{c} \end{aligned} \right.$ (Fig. S5).

The spatial distribution of dispersing flies from cell *c* to neighbouring cells ${Prob}_{c\to i\in\{v\}}$ was determined by the relative attractiveness of neighbouring cells $a_{t,i\in\{v\}}$ (Eq. S11-12). This attractiveness was designed to favour the emptiest cells and cells of greatest carrying capacity if similarly filled (i.e., $A_{t,i}\ll k_{i}$). An extended Moore neighbourhood of range $r$ was used: flies dispersed from a cell to its (2$r$ + 1)² neighbours (*v*), which included the cell itself and diagonals. Parameter $r$ is the maximum distance reached daily, in number of cells, rather than the effective distance covered per fly per day, as the trajectory is not linear. It was calibrated using data by taking into account the average $\frac{distance\left( m \right)}{time\left( days \right)}$ between release and capture of marked flies (Fig. S4).

$a_{t,i\in\{v\}}=\frac{\left( 1-exp\left( \frac{-k_{i}}{A_{t,i}} \right) \right)k_{i}}{max\left( k_{i\in\{v\}} \right)}$ (Eq. S11)

${Prob}_{c\to i\in\{v\}}=\frac{a_{i}}{\sum_{j\in v} a_{j}}$ (Eq. S12)

**
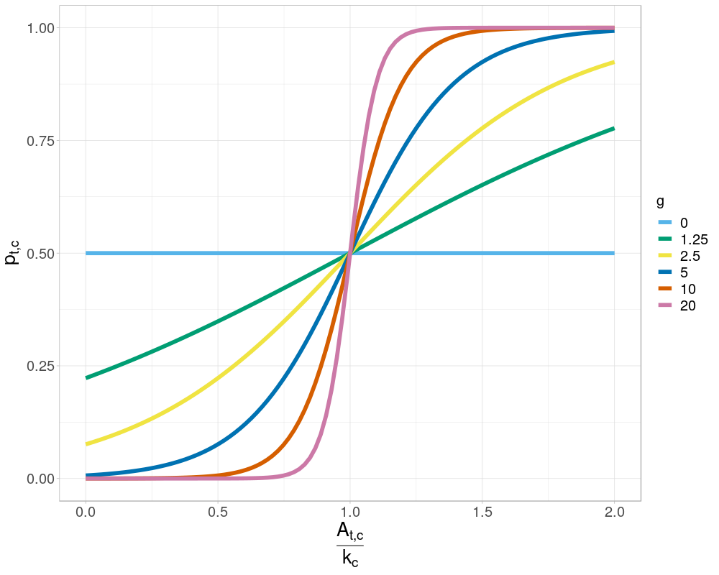
**

### **Fig. S5.** Dispersal rate $p_{t,c}$ as a function of $\frac{A_{t,c}}{k_{c}}$ and parameter $g$. The orange line is the equation used in the model (*g*=10).

## **4.3 Model setting**

A 3-year burn-in period was simulated starting with *N_0,c_*=*M_0,c_*=0.5*k_c_* (*A_0,c_*=*k_c_*), using reference parameter values (Table S2), and these provided the initial conditions for all the scenarios, and for the model sensitivity analysis. Population dynamics was simulated over three more years, except for control scenarios for which it was simulated over one year. Carrying capacities were spatially heterogeneous (Fig. S1C) but assumed constant over time. Daily perceived temperatures were estimated per cell for one year and these were repeated during the following years.

### **Table S2.** Parameter values. These parameters are used in the equations provided in the main text.

| Parameter | Symbol | Value | Standard error / [Reference] |
| --- | --- | --- | --- |
| Pupa mortality rate (/day) | m_P_ | 0.01 | [Childs 2011] |
| Mortality function parameters for parous females | m_1,F_ | 0.358 | 0.004 |
|  | m_2,F_ | -12.94 | 0.09 |
| Mortality function parameters for adult males | m_1,M_ | 0.254 | 0.007 |
|  | m_2,M_ | -9.53 | 0.19 |
| Development function parameters for pupae | d_1,P_ | 106.2264 | 7.5891 |
|  | d_2,P_ | 18.9483 | 5.2338 |
|  | d_3,P_ | 20.2279 | 0.3261 |
|  | d_4,P_ | 2.4901 | 0.7479 |
| Development function parameters for nulliparous females | d_1,N_ | 0.0020 | 0.0009 |
|  | d_2,N_ | 0.061 | 0.002 |
| Development function parameters for parous females | d_1,F_ | 0.0052 | 0.0001 |
|  | d_2,F_ | 0.1046 | 0.0004 |
| Shape parameter of the dispersal function | g | 10 | * |
| Cell carrying capacities [min, med, max] | k_c_ | [6.64 ; 145.65 ; 6547.48] | ° |
| Cell daily temperatures (°C) [min, med, max] | θ_t,c_ | [18.66 ; 24.34 ; 29.90] | ° |

*To the best of our knowledge; °Data-driven

## **4.4 Additional results on the scenario without control**

**Accounting for spatial dispersal**

**
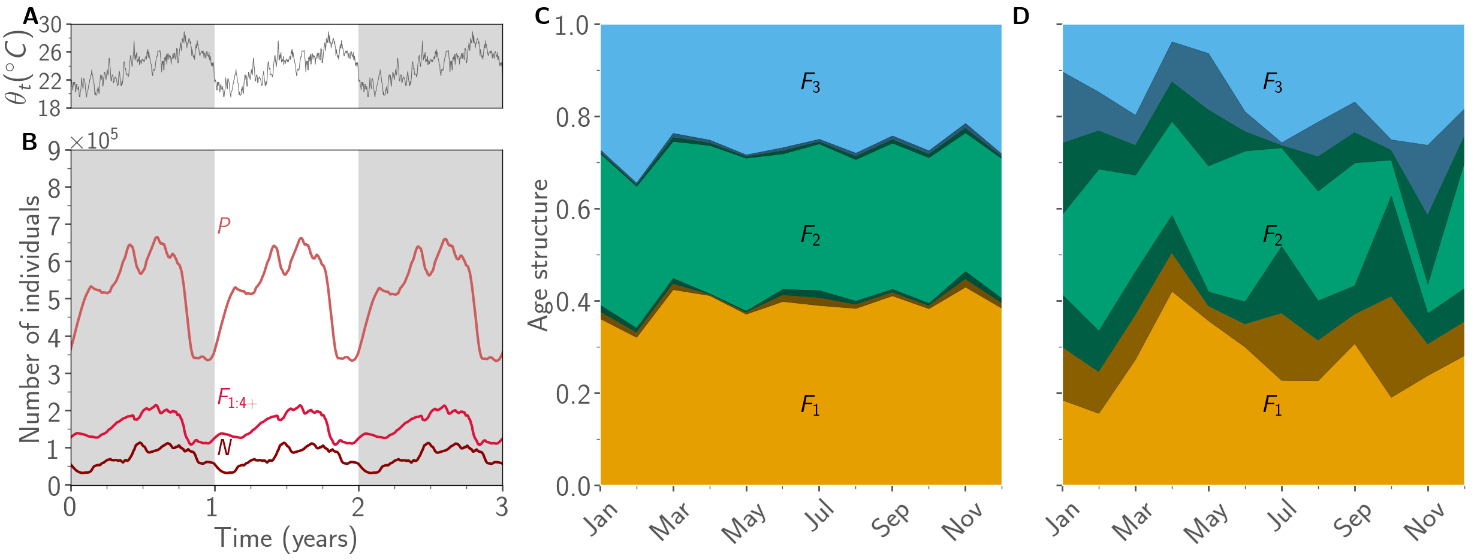
**

**Neglecting fly dispersal**

**
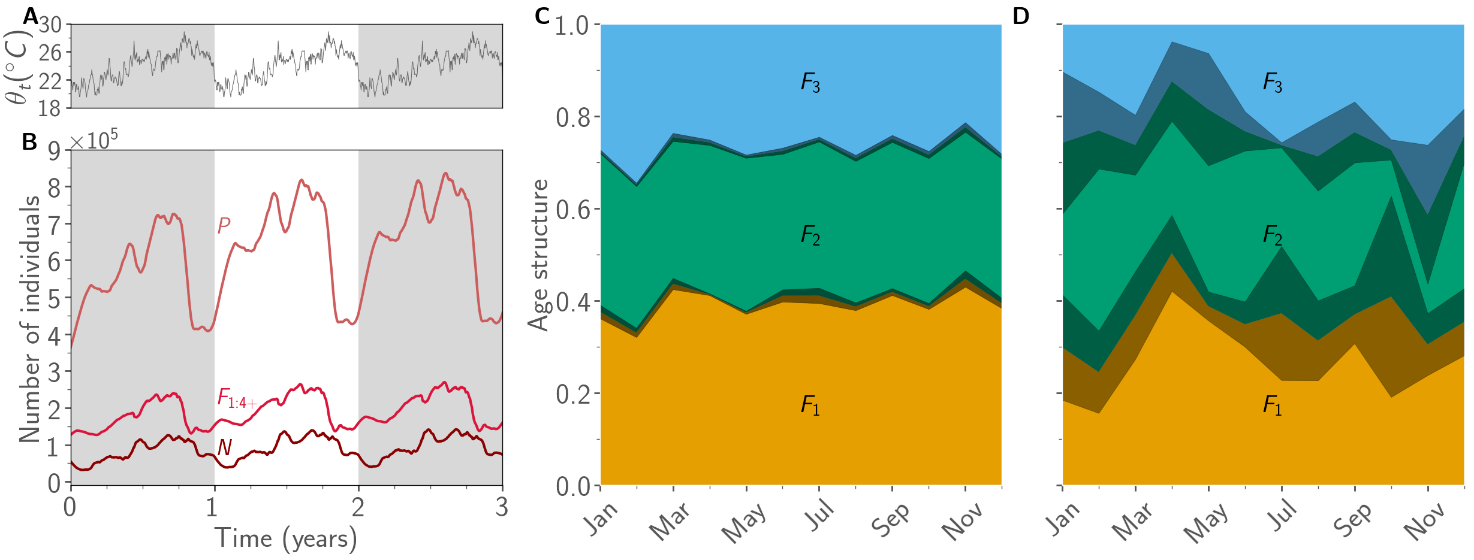
**

### **Fig. S6.** Model predictions for the scenario without control. First line: reference model accounting for fly spatial dispersal. Second line: model neglecting fly dispersal. Per line: A: average daily temperatures over three years (in °C); B: total number of individuals per stage (P: pupae, N: nulliparous females, F: parous females) in the grid (56.25 km^2^) over three years of simulation; C: female age structure ($\frac{F_{i=1,2,3}}{F_{1}+F_{2}+F_{3}}$) during the last year of simulation; D: observed female age structure (captures and dissection occurred from 2008 to 2011 in the Niayes; results were averaged by month, all years and locations aggregated; grey filled areas are confidence intervals around the mean: $\frac{\pm t_{5\%}^{n_{month}-1}\times{sd}_{month}}{\sqrt{n_{month}}}$, with $t_{5\%}^{n_{month}-1}$ the 5^th^ centile of Student low with *n_month* – 1 degree of freedom, *sd_month_* the standard deviation and *n_month_* the number of measures, i.e. the number of days in the month for simulations, the number of captures for data).

**
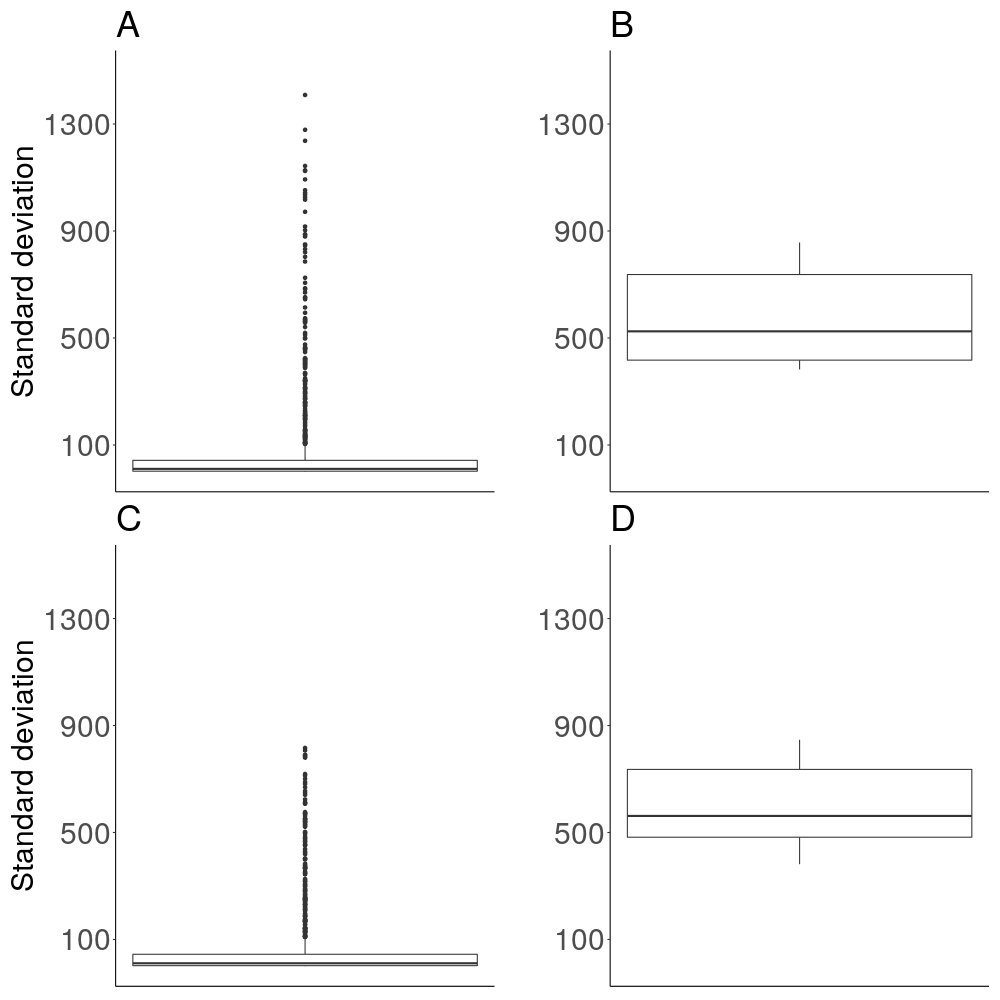
**

### **Fig. S7.** Variability of tsetse fly population size. A: within cell over time, reference model; B: between cell in space, reference model; C: within cell over time, neglecting dispersal; D: between cell in space, neglecting dispersal.

# 5. Sensitivity analysis

The individual and joint effects of input variations on aggregated output variance (Table S3) were evaluated through a variance-based global sensitivity analysis using the Fourier Amplitude Sensitivity Testing (FAST) method (Saltelli et al. 2008).

### **Table S3.** Definition of aggregated outputs.

| **Output** | **Description** | **Comment** |
| --- | --- | --- |
| popMean | Average female population size (N+F_1:4+_) over the year in the grid | Results in main text and Fig. S9 |
| popStd | Standard deviation of female population size (N+F_1:4+_) over the year in the grid |  |
| surfX | Average proportion of cells in the study area with $\frac{\bar{A_{c}}}{k_{c}}\geq X$ ($\bar{A_{c}}$: average number of adults (nulliparous and parous females, males) over the year in cell *c*) | X = [10,20,50,75,80,90] % |
| surf1 | Average proportion of cells containing at least one adult (nulliparous and parous females, males) on average |  |
| miX, meX, maX | Minimum, mean, maximum $\frac{F_{x}}{F_{1}+F_{2}+F_{3}}$ over the grid and over the year (%) | X = [1,2] |
| mean_distrib | Average $\frac{\bar{A_{c}}}{k_{c}}$ over the year |  |
| std_distrib | Standard deviation of $\frac{\bar{A_{c}}}{k_{c}}$ over the year |  |
| qX_distrib | Percentile X of $\frac{\bar{A_{c}}}{k_{c}}$ over the year | X = [5,25,75,95] |
| median_distrib | Median of $\frac{\bar{A_{c}}}{k_{c}}$ over the year |  |

As traps do not catch nulliparous and females of ovarian age 4 and more as efficiently as females of intermediate ovarian ages (Saunders 1962), predicted age structure was compared with field data for females of ovarian age 1, 2, and 3: $\frac{F_{i=1,2,3}}{F_{1}+F_{2}+F_{3}}$. Mortality and development functions of each life stage were varied using multiplying factors (i.e. function formulas were kept). The reference values of multiplying factors were all equal to one. A common factor was applied to all adult mortalities (*N*, *M*, *F_1:4+_*) to maintain a similar order of values. A multiplying factor was also applied to carrying capacities to regulate the magnitude of density-dependence. As the dispersal rate should remain in the range [0-1], the shape parameter *g* was varied (Fig. S5). Parameters and multiplying factors were varied by ± 5% of their reference value. The same range, when applied to temperature, changed the annual mean by more than 2°C, which was far greater than what was observed. Therefore, a variation of ± 0.3°C was used, corresponding to the average deviation from the daily mean in the area (Fig. S8). First order and interaction sensitivity indices were calculated per parameter (Saltelli et al. 2008). Each parameter set (scenario) was applied in simulations of 1 year.

###
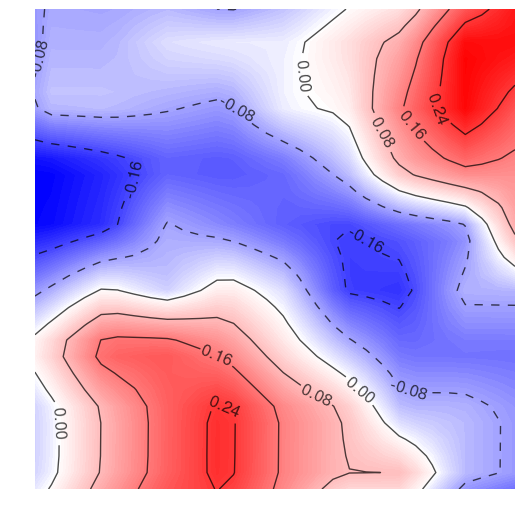
**Fig. S8.** Scaling down of temperature variations for the global sensitivity analysis. Average $T_{cell}-{mean}_{day}\left( T \right)$ = $\frac{\sum_{t} \left( \theta_{t,c}-\frac{\sum_{i} \theta_{t,i}}{30*30} \right)}{365}$over the year for every cell. Maximum is +0.3°C.

**
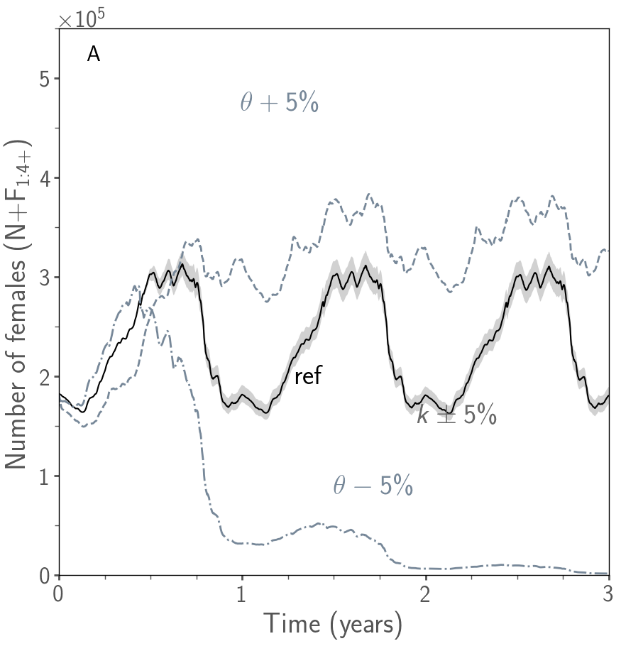
**

### **Fig. S9.** Sensitivity of the predicted population size to variations in temperature (+5%: dash dot, -5%: dashed) and carrying capacity (±5%, grey filling).


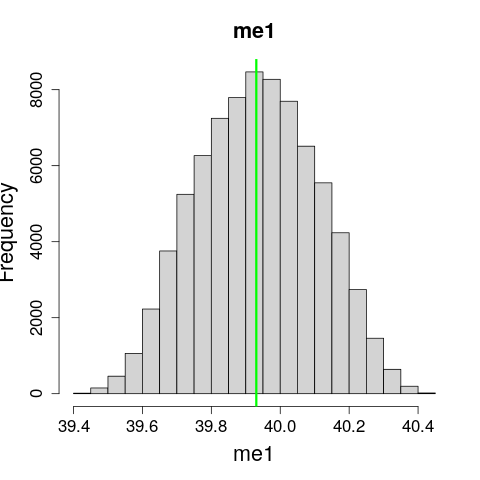

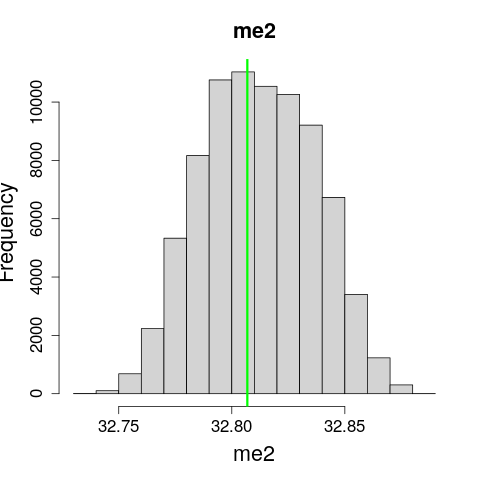


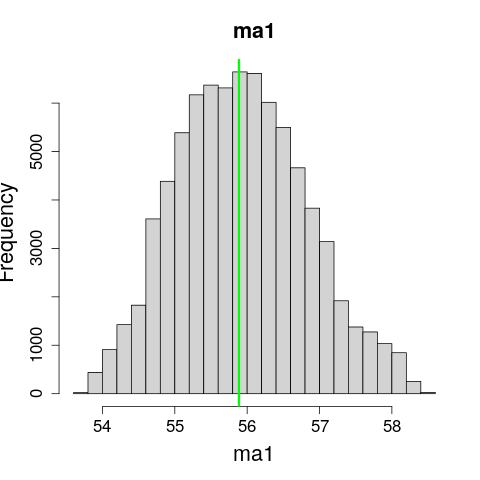

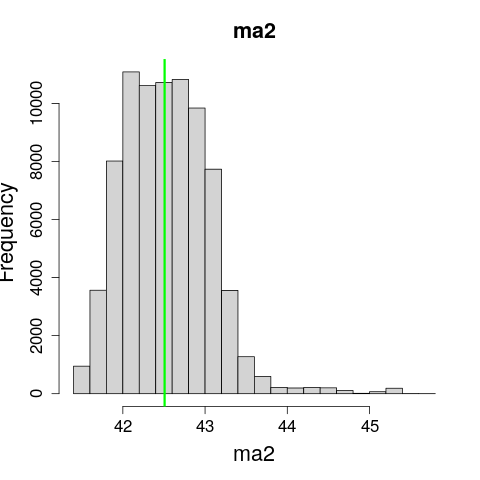


###
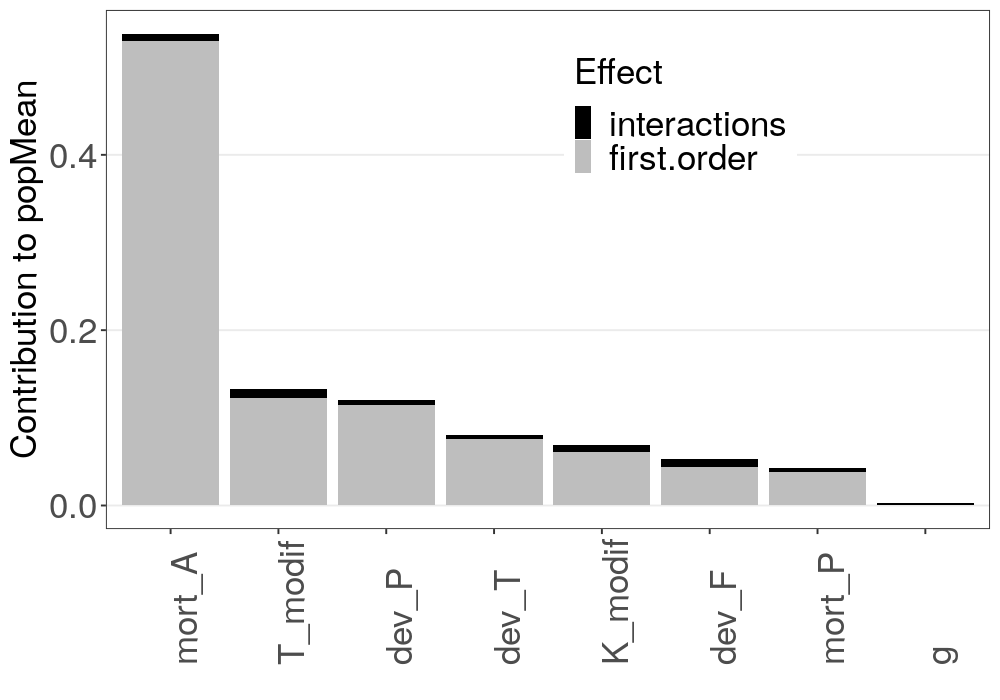

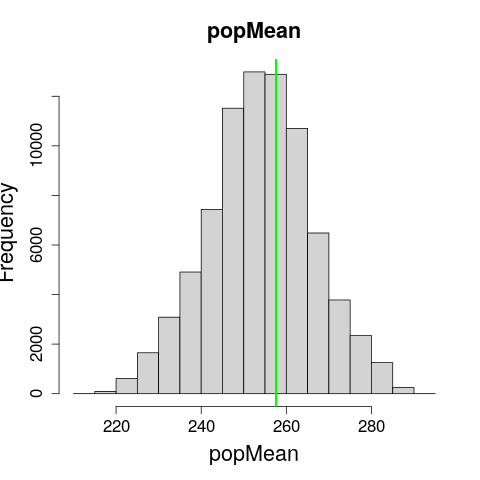
**Fig. S10.** Age structure showed almost no variation with parameter variations, thus was not further analysed. Here, we show variations in the mean (Me) and maximum (Ma) proportions of females of parity *X* ($\frac{F_{x}}{F_{1}+F_{2}+F_{3}}$, *X* in [1-2]) over the grid the last year of simulation. Green line: no-control scenario. All parameters varied by ±5% from their reference value except temperature varying by ±0.3°C.

μ_{N,F,M}_ θ δ_P_ δ_N_ k δ_F_ μ_P_ g


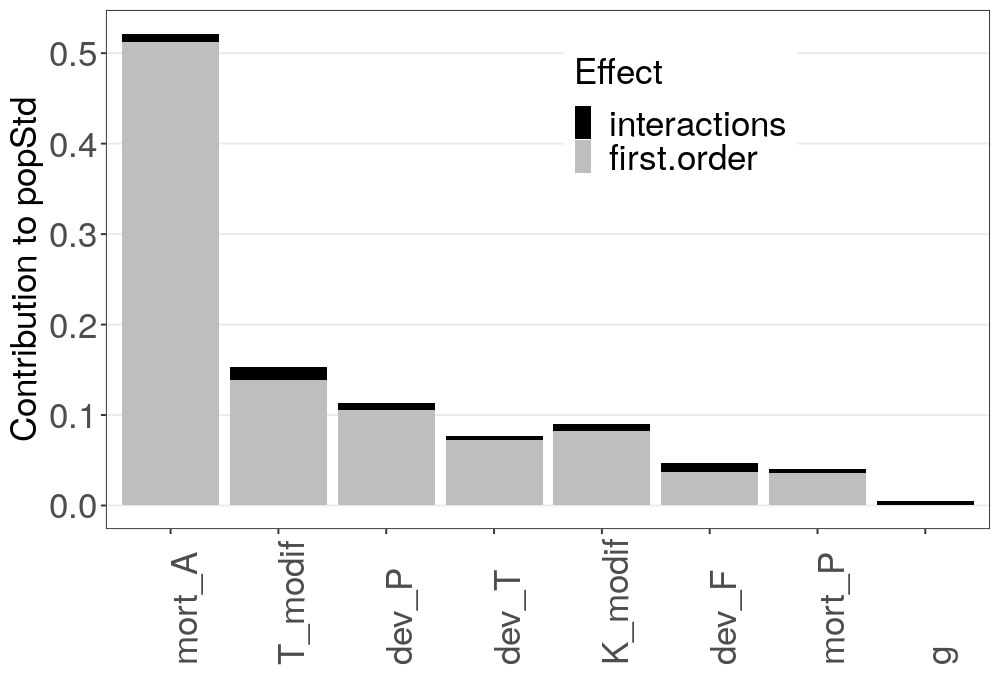

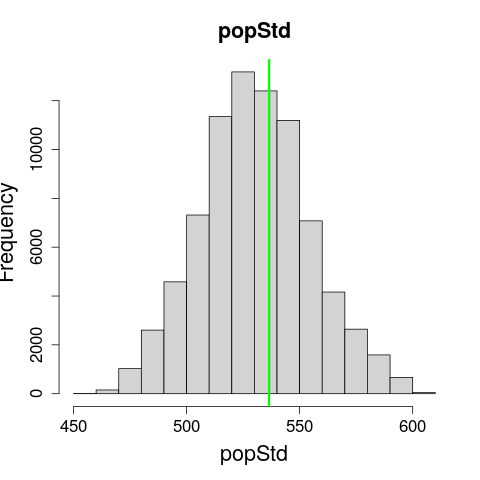


μ_{N,F,M}_ θ δ_P_ δ_N_ k δ_F_ μ_P_ g


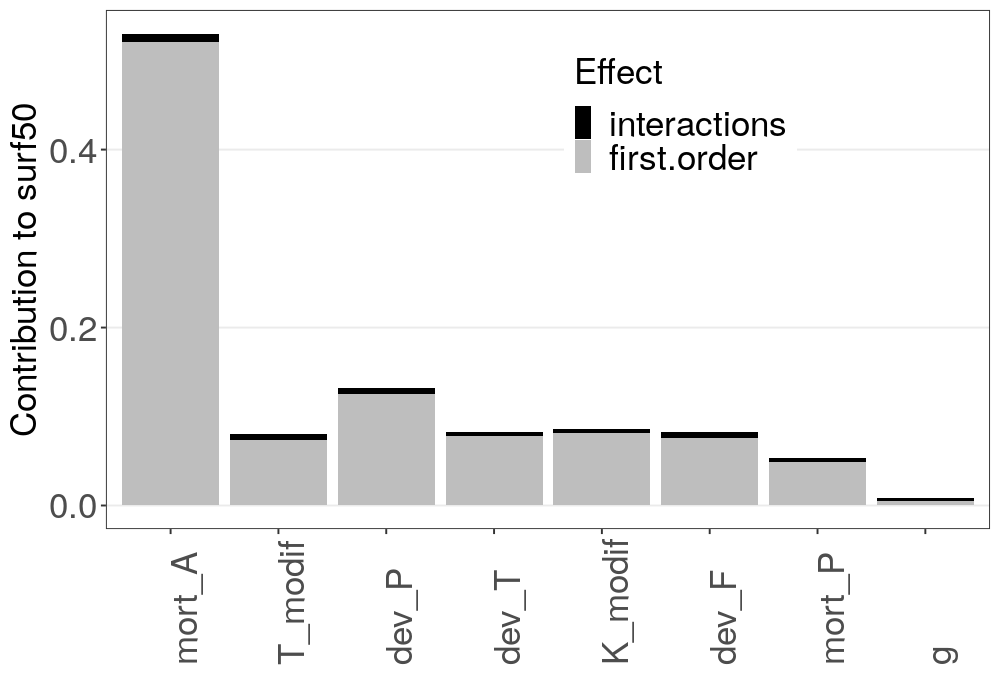


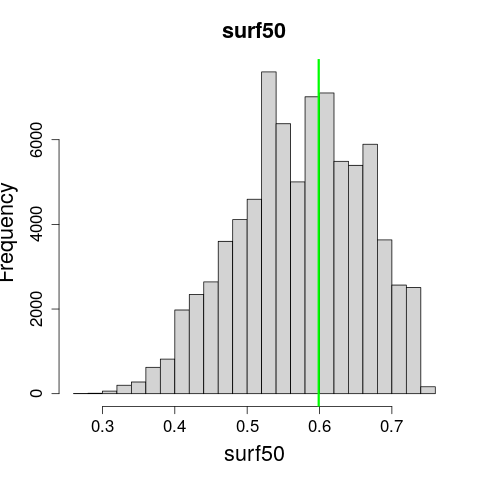


μ_{N,F,M}_  θ δ_P_ δ_N_ k δ_F_ μ_P_ g


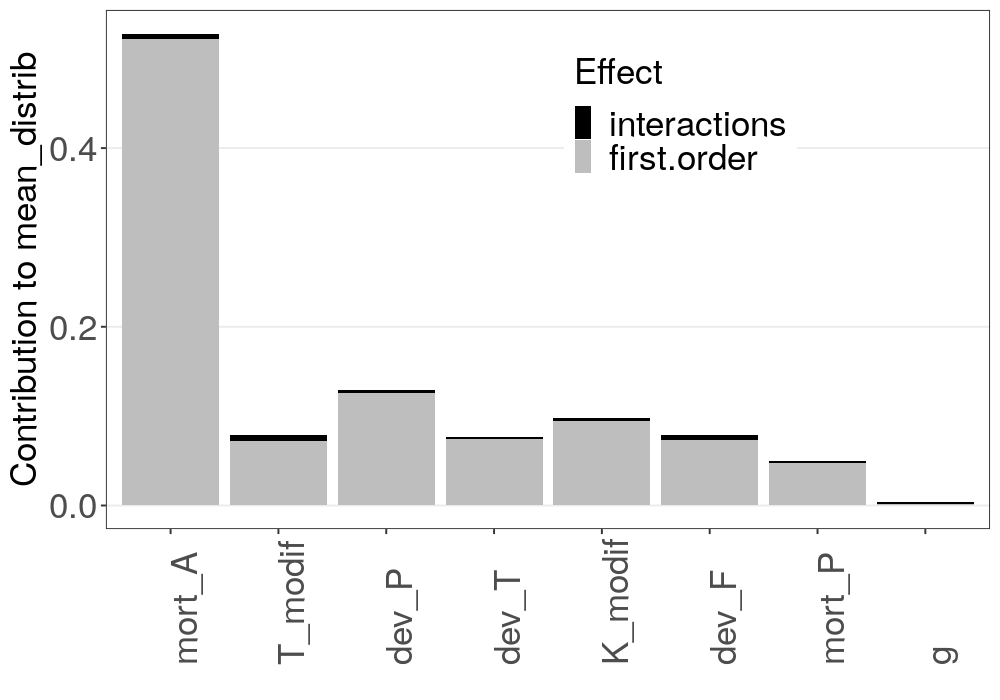

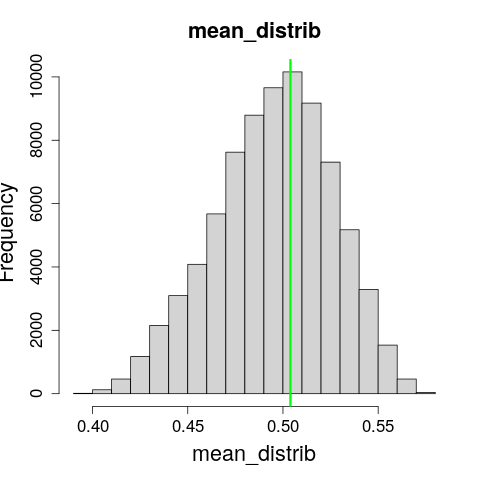


μ_{N,F,M}_ θ δ_P_ δ_N_ k δ_F_ μ_P_ g


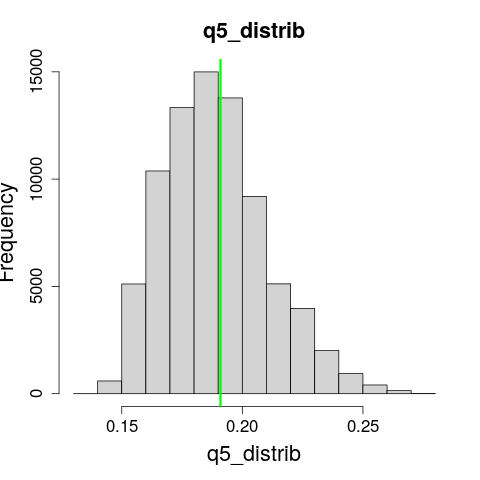

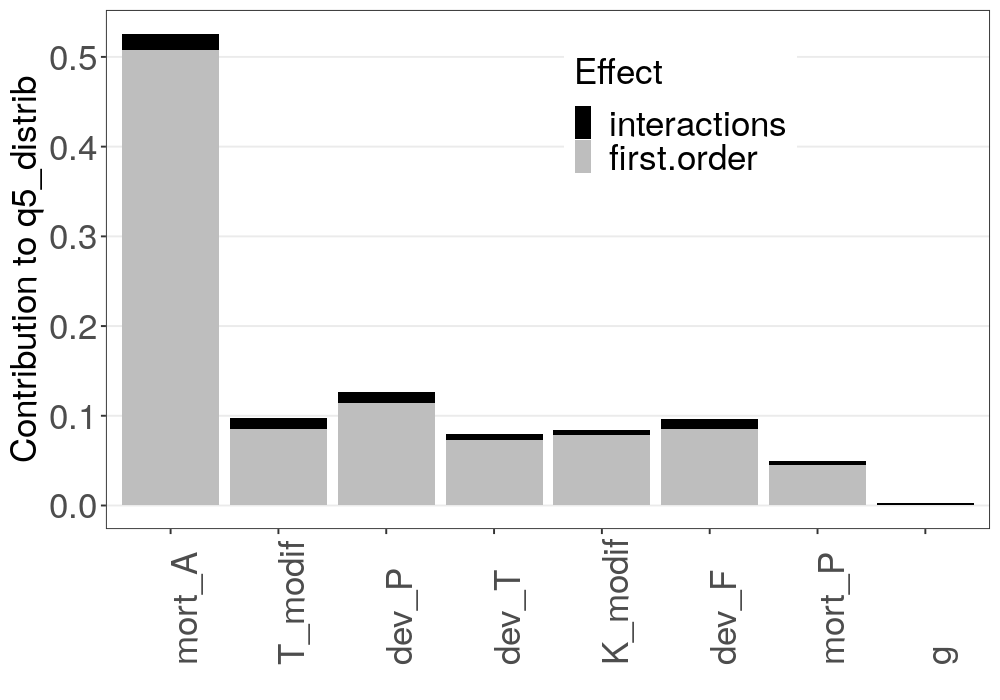


μ_{N,F,M}_ θ δ_P_ δ_N_ k δ_F_ μ_P_ g


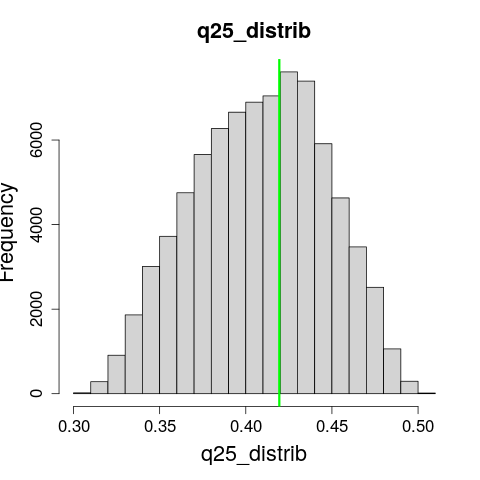

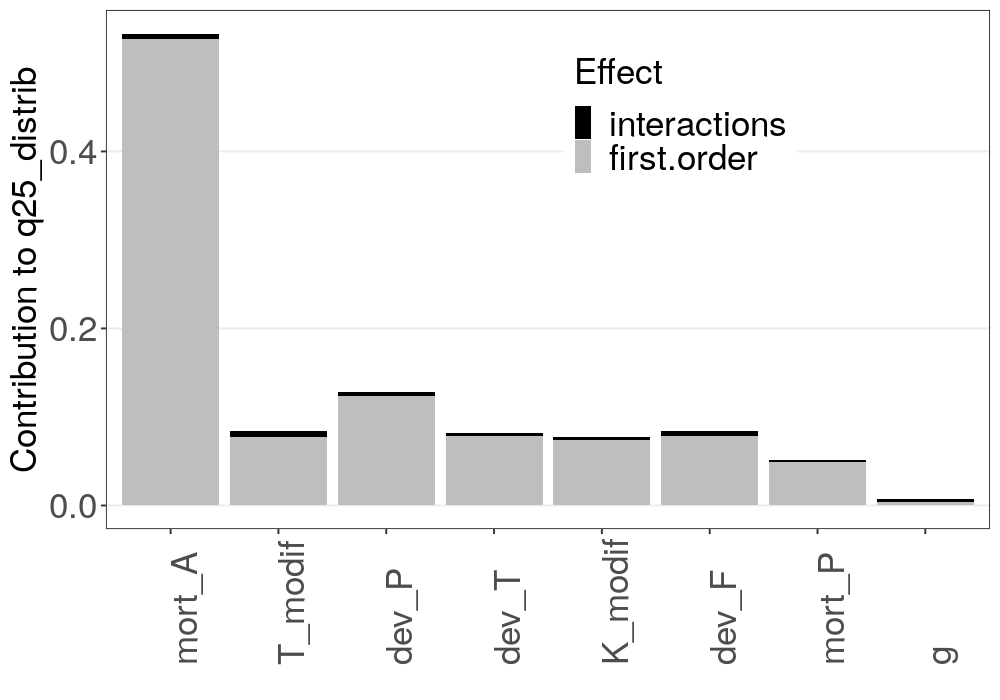


μ_{N,F,M}_ θ δ_P_ δ_N_ k δ_F_ μ_P_ g


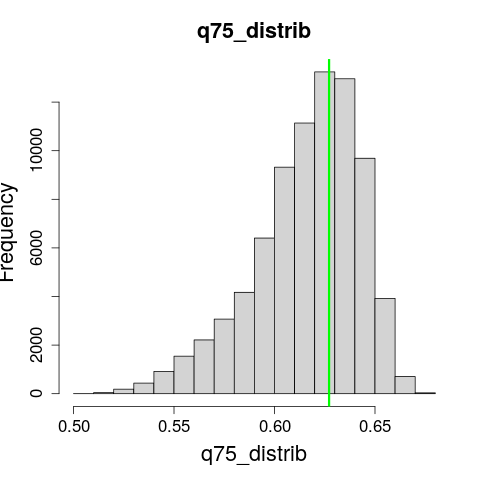

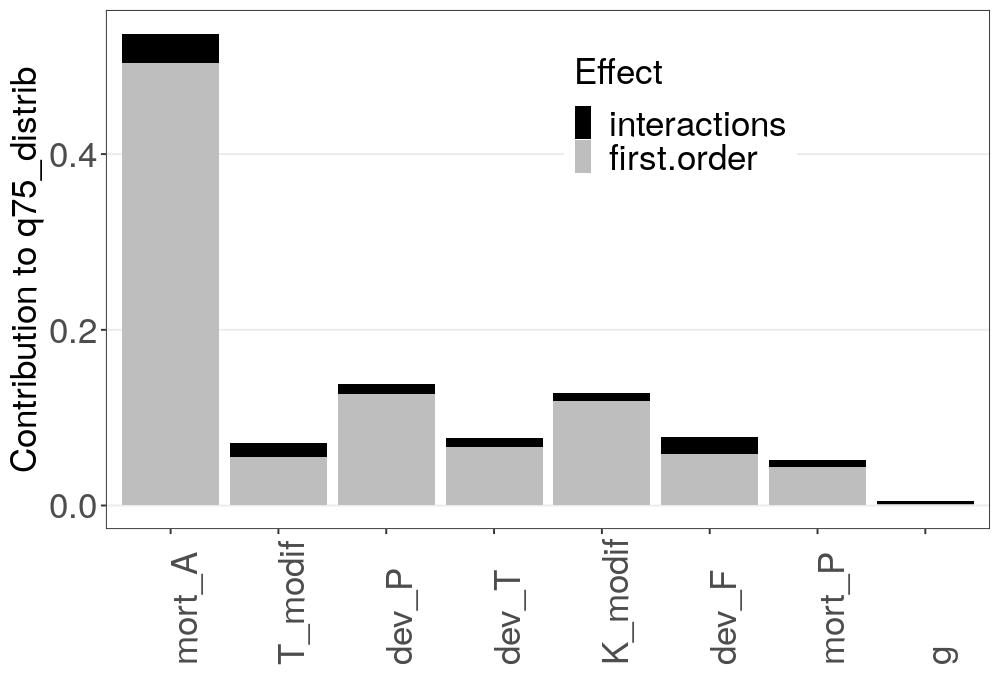


μ_{N,F,M}_ θ δ_P_ δ_N_ k δ_F_ μ_P_ g


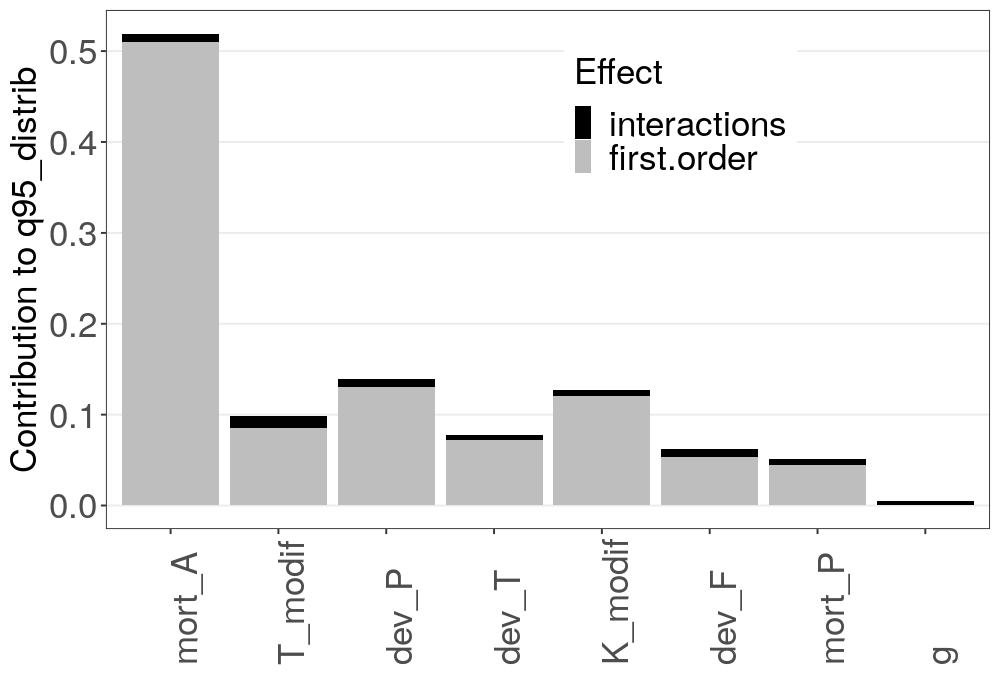

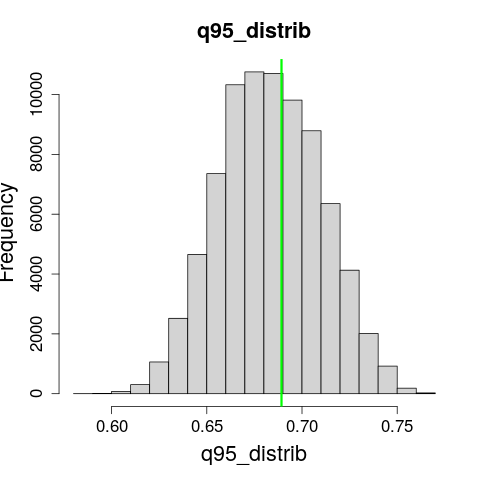


μ_{N,F,M}_ θ δ_P_ δ_N_ k δ_F_ μ_P_ g


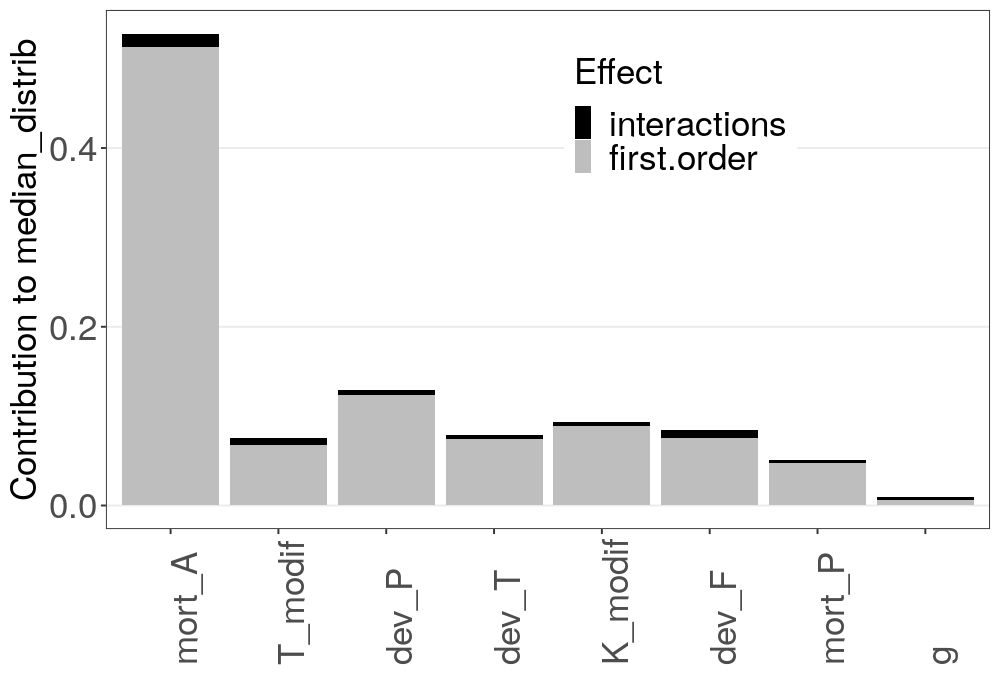

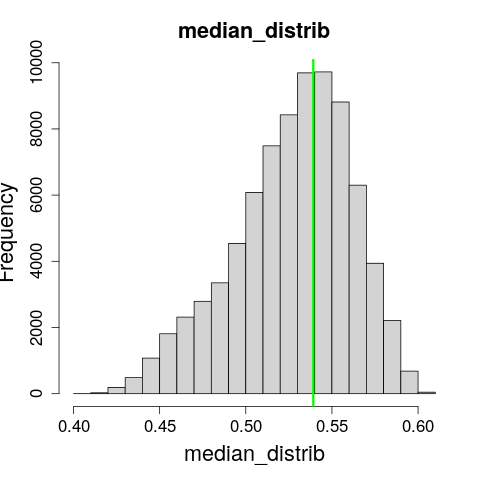


μ_{N,F,M}_ θ δ_P_ δ_N_ k δ_F_ μ_P_ g

### **Fig. S11.** Global sensitivity analysis. Left: output distributions. Right: principal effect (in grey) and interaction (in black) sensitivity indices for each output (refer to Table S3 for output definition) per varying parameter ((*θ*: temperature, *µ_{N,F,M}_*: adult mortality, δ_X_: time to development of stage *X* (*P*: pupae, *N*: nulliparous females, *F*: adult females), *k*: carrying capacity, *g*: shape parameter in the diffusion process). Green line: no-control scenario. All parameters varied by ±5% from their reference value except temperature varying by ±0.3°C.

# 6. Additional information about control strategies

A spatially targeted control strategy was mimicked by increasing female mortality during one year, starting from the same initial conditions as in the scenario without control. For successively reduced proportions of controlled cells (starting with 100% of the cells being controlled), we assessed the minimal mortality increase needed to decrease the female population size down to 2% (respectively 5%) of its initial size over the whole grid after one year. We stopped reducing the proportion of controlled cells once it became impossible to achieve the targeted population reduction whatever the mortality rate.

We did not test all combinations of controlled cells for all proportions. To minimize the number of scenarios to be tested, the proportion of controlled cells gradually decreased while mortality gradually increased. For a given proportion (x%), the cells to be controlled were selected from those also controlled for the scenario with the next highest proportion tested (y%>x%). The cells removed from treatment were preferentially those where the treatment had been least effective in the previous scenario (“y%”) using the score defined thereafter. Mortality was then gradually increased until the female population was reduced to 2 or 5% of its original size. If this was not possible, then a lower proportion of cells to be controlled was not tested further.

We defined a score to optimize the selection of controlled cells. The best location of controlled cells was defined by assessing the contribution of each cell *j* to the total female population over the grid (*n* cells) at the end of the control (*t* = 1 year) if cell *j* was not controlled (Eq. S13):

$P_{j}=\sum_{i=1}^{i=j-1} T_{t=1 yr,i}^{control}+\sum_{i=j+1}^{i=n} T_{t=1 yr,i}^{control}+T_{t=1 yr,j}^{no control}$ (Eq. S13)

where the total number of females in cell *c* was: $T_{t=1 yr,c}=N_{t=1 yr,c}+F_{1:4+,t=1 yr,c}$.

Cells with the highest *P_j_* were given priority control. As a result, optimized strategies were defined by the minimal proportion of cells to be controlled, their optimal location, and the control effort required.

The control efficacy was assessed with respect to the female population size at both grid and cell scales. We computed for each cell *c* after one year of control: (1) the proportion of females in the area which were located in that cell, $\frac{T_{t=1 yr,c}}{\sum_{i} T_{t=1 yr,i}}$, which indicated cells with the highest proportion of the female population; (2) the abundance of females in cell *c* in the control vs. the no-control scenarios after one year $\frac{\left( T_{t=1 yr,c} \right)_{control}}{\left( T_{t=1 yr,c} \right)_{no-control}}$, which quantified the local impact of increased mortality.

Then, population resurgence was simulated for one more year after the end of the control period, taking into consideration the reference value of female mortality. To identify the cells that contributed most to the recovery of the population, the local growth rate was calculated per cell: $\frac{T_{t=2 yr,c}-T_{t=1 yr,c}}{T_{t=1 yr,c}}$, $T_{t=2 yr,c}$ being the female abundance in cell *c* one year after the end of the control period (t = 2 years).

We analysed the relationships between the local environmental variables (carrying capacity, mean temperature, temperature variance in each cell) and these three cell indicators, reflecting different properties of the population spatial structure.

###
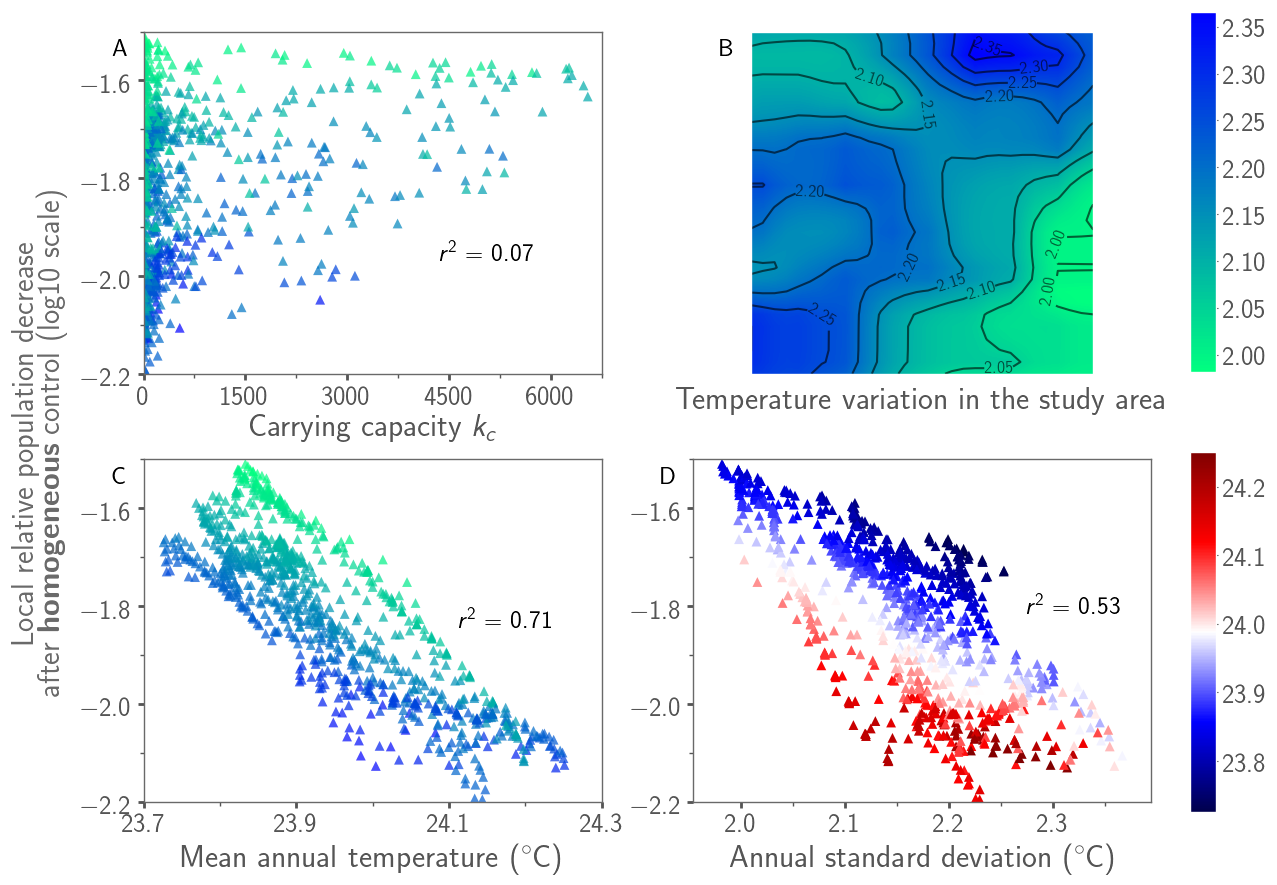


### **Fig. S12.** Local efficacy of a homogeneous control applied in all cells (female life expectancy of 35 days on average), with the lowest values of the relative population decrease (y-axis) representing the highest control efficacy. A: no correlation was observed with the local carrying capacity (A). B: spatial representation of the annual standard deviation of local temperatures (the colour bar was also used in A and C). C: the mean annual temperature was correlated with local efficacy. D: the annual standard deviation of temperature was correlated with local efficacy (points coloured by mean temperature to check the absence of correlation). Each point corresponds to a cell of the simulated grid.


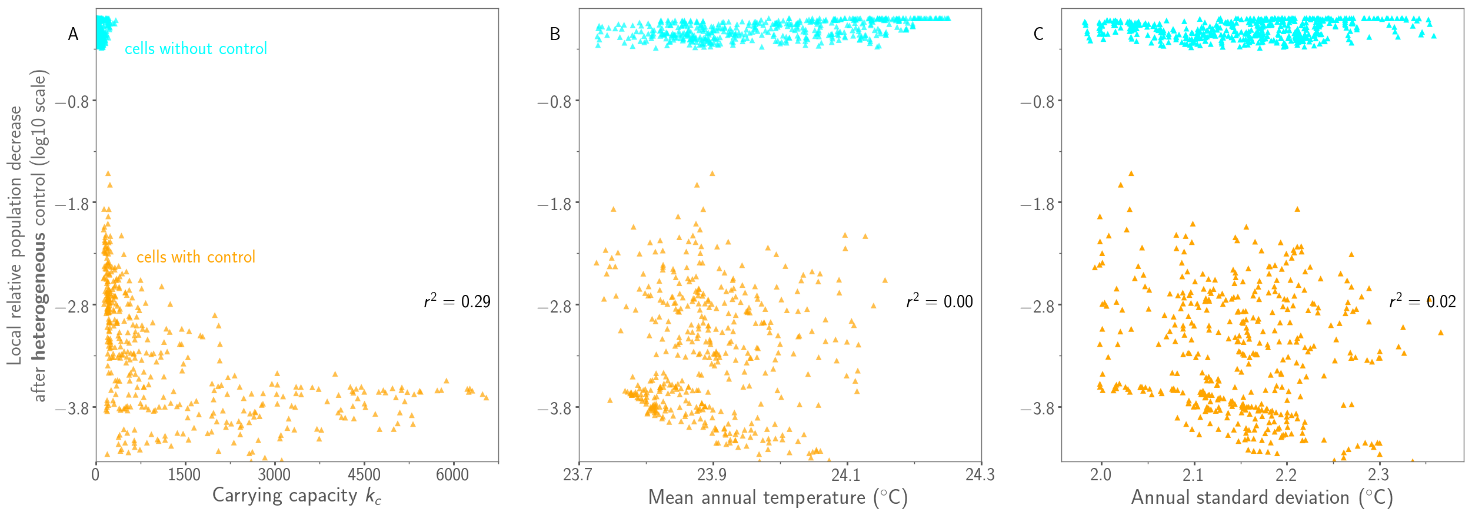


### **Fig. S13.** Local efficacy of a heterogeneous control applied in 47% of the cells (orange: treatment inducing a female life expectancy of 23 days on average; cyan: no treatment, female life expectancy of 60 days on average). No correlation was observed with local cell variables (A: carrying capacity; B: mean annual temperature; C: annual standard deviation of temperature).

# 7. Additional results when neglecting fly spatial dispersal


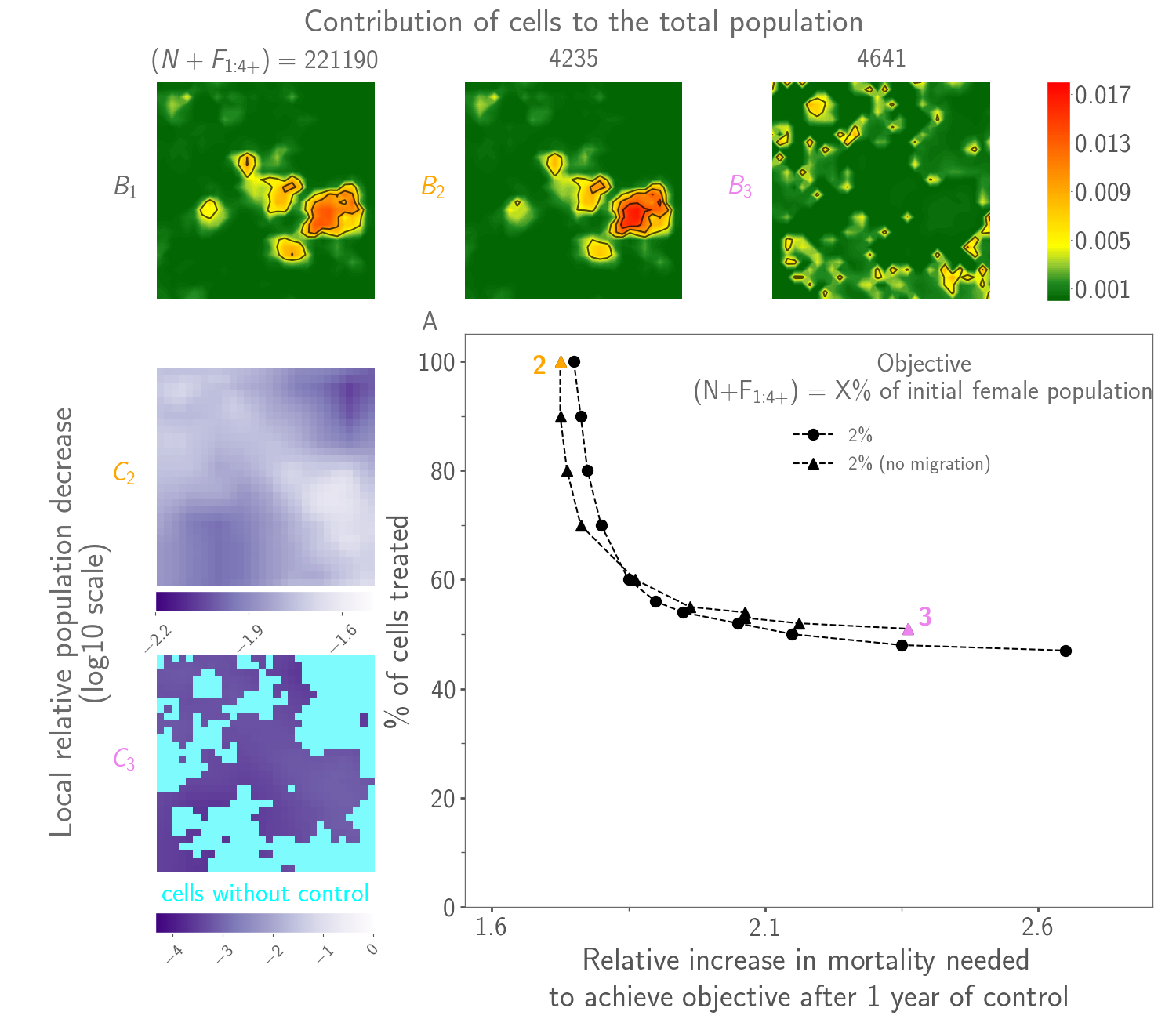


### **Fig. S14. To be compared to Fig. 2.** Mortality increase and tsetse fly population size when neglecting fly spatial dispersal. A: relative increase in mortality needed to reduce the female population size to 2% (circle) or 5% (triangle) of its initial size after one year of control, when a fraction of cells was targeted. B: contribution of cells to the total population size (1: no control, 2: homogeneous control, 3: heterogeneous control targeting 51% of the cells). C: local control efficacy (2-3: same as in B), the darkest being the most effective (cyan: uncontrolled cells).


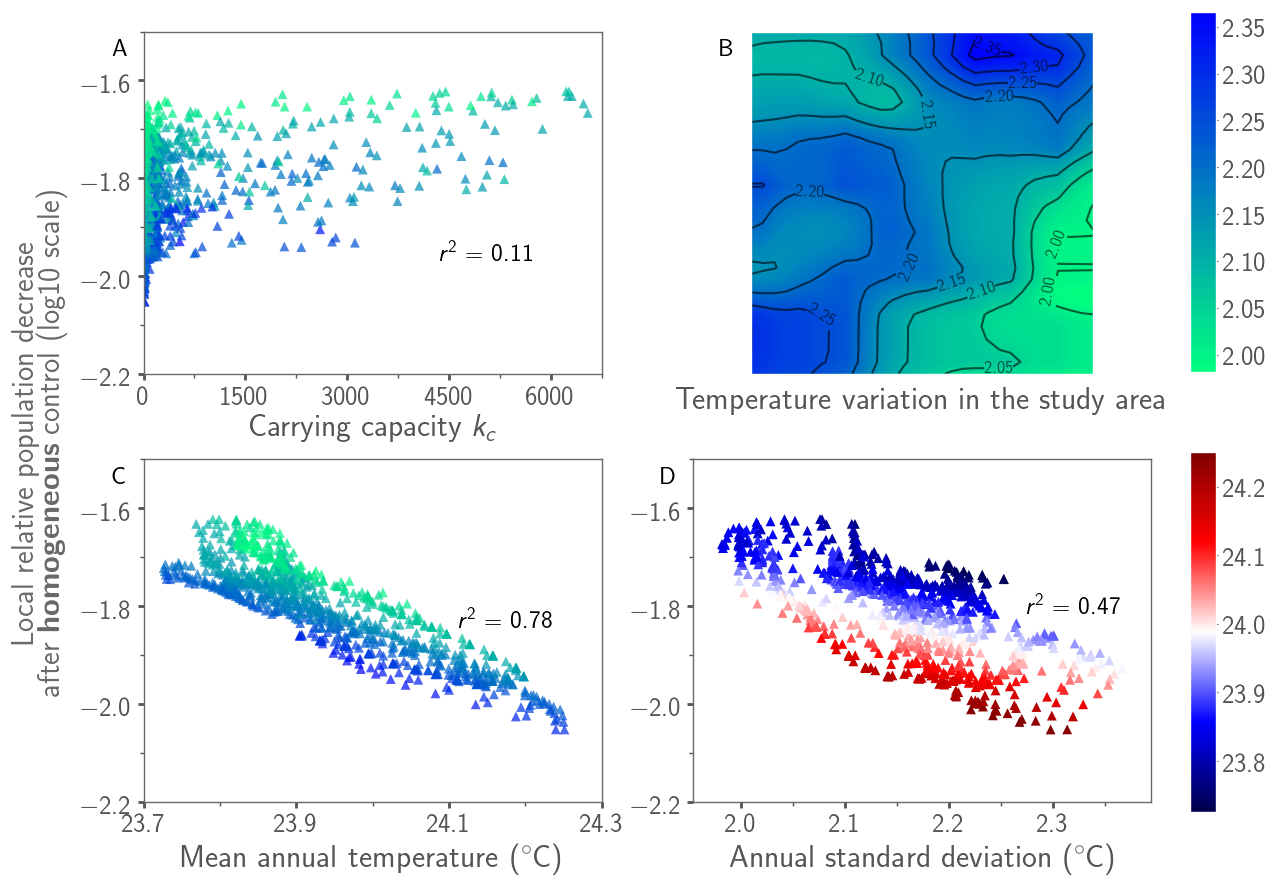


### **Fig. S15. To be compared to Fig. S12.** Local efficacy of a homogeneous control applied in all cells when neglecting fly dispersal (female life expectancy of 35 days), with the lowest values of the relative population decrease (y-axis) representing the highest control efficacy. A: no correlation was observed with the local carrying capacity (A). B: spatial representation of the annual standard deviation of local temperatures (the colour bar was also used in A and C). C: the mean annual temperature was correlated with local efficacy. D: the annual standard deviation of temperature was correlated with local efficacy (points coloured by mean temperature to check the absence of correlation). Each point corresponds to a cell of the simulated grid.


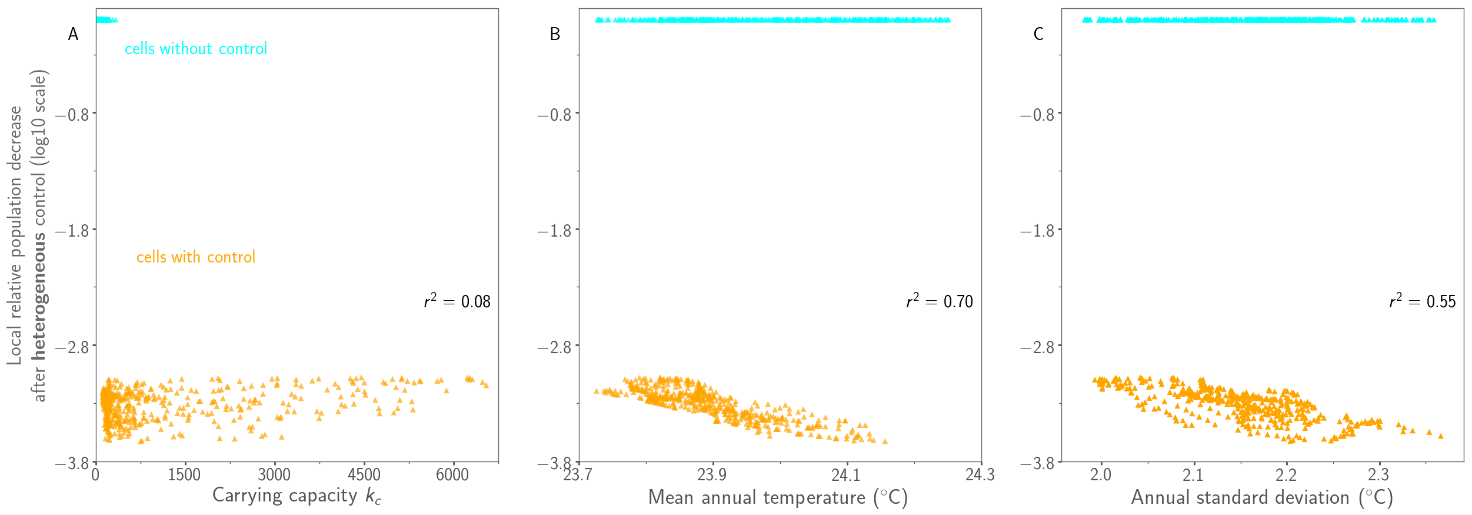


### **Fig. S16. To be compared to Fig. S13.** Local efficacy of a heterogeneous control applied in 51% of the cells when neglecting fly dispersal (orange: treatment inducing a female life expectancy of 26 days on average; cyan: no treatment, female life expectancy of 60 days on average). No correlation was observed with the carrying capacity (A), but correlations were observed with the mean annual temperature (B) and the annual standard deviation of temperature (C).


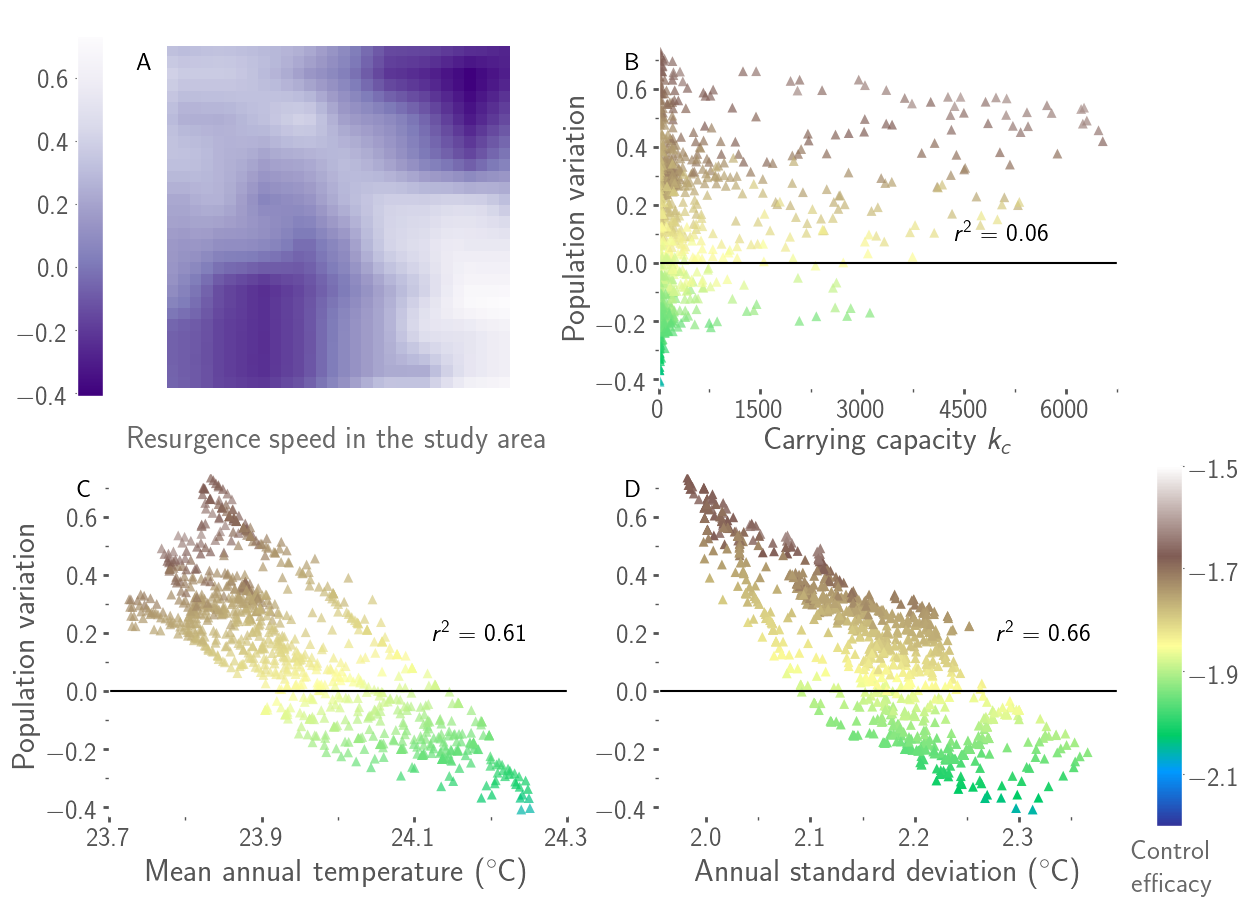


### **Fig. S17. To be compared to Fig. 3.** Local population resurgence one year after the end of a homogeneous control when neglecting fly dispersal. A: local growth rate. B-D: variations of the local growth rate with carrying capacity, mean annual temperature, and annual standard deviation of temperature. Colours: control efficacy (blue: most effective).


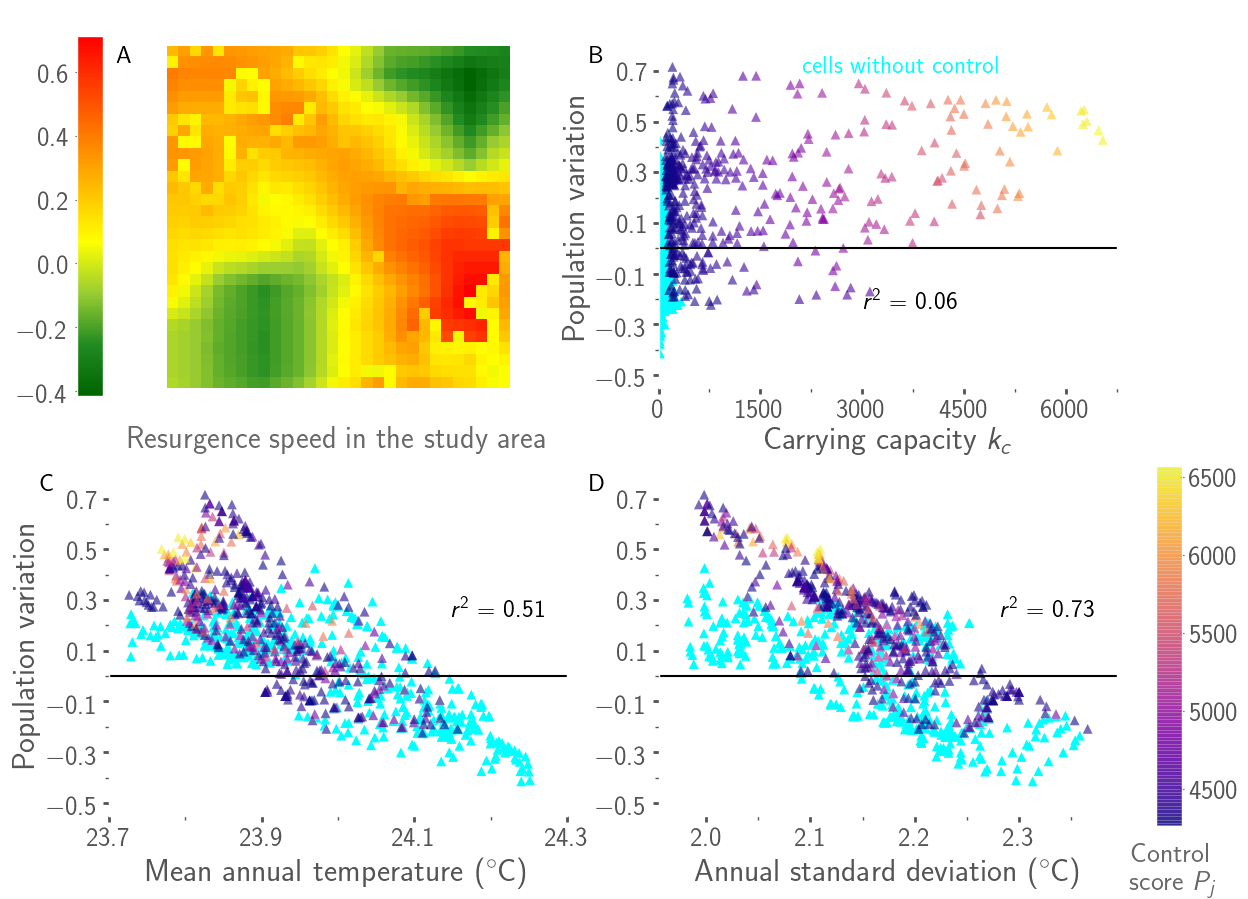


### **Fig. S18. To be compared to Fig. 4.** Local population resurgence one year after the end of a heterogeneous control (51% of controlled cells) when neglecting fly dispersal. A: local growth rate. B-D: variations of the local growth rate with carrying capacity, mean annual temperature, and annual standard deviation of temperature. Colours: control score (the highest: still targeted when the proportion of controlled cells is reduced). Cyan: uncontrolled cells.

# 8. Code sources and data files

To use the model code, go to: <https://sourcesup.renater.fr/projects/spatial-tsetse/> or use directly the following command “git clone <https://git.renater.fr/anonscm/git/spatial-tsetse/spatial-tsetse.git>”. We also provide there in folder “input/data_calibration/” the following files:

- capture-mark-release (CMR) data used for calibrating dispersion (“dispersal/Captures.csv”, “dispersal/Releases.csv”);
- trap catch data used for assessing apparent density (“daily_apparent_density/dap_Niayes_Glossina_palpalis_gambiensis.csv”);
- data on physiological age (“age_structure/ovarian_age_from_capture.csv”);
- mortality data (“mortality/colony_maintenance_SN_strain_24_degrees.csv”, “mortality/mortality_temperature_lab_data.csv”);
- data on pupal duration (“pupal_duration/pupae_data_formatted_calibration.csv”, “pupal_duration/pupae_raw_data.ods” the latter being an Open Office file with several sheets).

# References

Alderton S, Macleod ET, Anderson NE, Schaten K, Kuleszo J, Simuunza M. et al. 2016 A Multi-Host Agent-Based Model for a Zoonotic, Vector-Borne Disease. A Case Study on Trypanosomiasis in Eastern Province, Zambia. PLoS Negl. Trop. Dis. 10(12):e0005252. https://doi.org/10.1371/journal.pntd.0005252

Bouyer J, Balenghien T, Ravel S, Vial L, Sidibé I, Thévenon S, et al. 2009 Population sizes and dispersal pattern of tsetse flies: rolling on the river? Mol Ecol. 18, 2787–2797. doi: 10.1111/j.1365-294X.2009.04233.x PMID: 19457176

Burnham KP, Anderson DR. 2002 Model selection and multimodel inference: a practical information-theoretic approach. 2nd ed. New-York: Springer-Verlag.

Challier A. 1965 Amélioration de la méthode de détermination de l’âge physiologique des glossines. Bull Soc Path Ex 58: 250–259.

Childs SJ. 2011 Theoretical levels of control as a function of mean temperature and spray efficacy in the aerial spraying of tsetse fly. Acta Tropica 117, 171–182.

Cressie NAC, Cassie NA. 1993 Statistics for spatial data. Wiley New York.

Dicko AH, Lancelot R, Seck MT, Guerrini L, Sall B, Lo M. et al. 2014 Using species distribution models to optimize vector control: the tsetse eradication campaign in Senegal. Proc. Nat. Acad. Sci. 111, 10149-10154.

Dicko AH, Percoma L, Sow A, Adam Y, Mahama C, Sidibé I. et al. 2015 A Spatio-temporal Model of African Animal Trypanosomosis Risk. PloS Negl. Trop. Dis. 9:e0003921.

Elith J, Phillips SJ, Hastie T, Dudík M, Chee YE, Yates CJ. 2011 A statistical explanation of MaxEnt for ecologists. Divers Distrib. 17, 43–57.

Hargrove JW. 2004 Tsetse population dynamics. In: The Trypanosomiases. Ed. by I. Maudlin, P. Holmes, and P. Miles. Oxford, UK: CABI Publishing, pp. 113–137.

Kilibarda M, Hengl T, Heuvelink GBM, Gräler B, Pebesma E, Perčec Tadić M. et al. 2014 Spatiotemporal interpolation of daily temperatures for global land areas at 1 km resolution. J. Geophysical Res.: Atmospheres 119, 2294–2313.

Laveissière C, Grébaut P. 1990 Recherches sur les pièges à glossines (Diptera, Glossinidae). Mise au point d'un modèle économique : le piège "Vavoua". Trop. Med. Parasitol. 41: 185-192.

Lloyd-Smith JO. 2010 Modeling density dependence in heterogeneous landscapes: Dispersal as a case study. J. Theor. Biol. 265, 160–166.

Pagabeleguem S. 2012 Etude de compétitivité des mâles stériles dans le cadre de l’utilisation de la technique de l’insecte stérile pour l’éradication des glossines dans la zone des Niayes au Sénégal. Univ. Montpellier II, France – Univ. Abomey Calavi, Bénin. p. 31.

Pagabeleguem S, Seck MT, Sall B, Vreysen MJB, Gimonneau G, Fall AG, Bassene M, Sidibé I, Rayaisse JB, Belem A, Bouyer J. 2015 Long distance transport of irradiated male Glossina palpalis gambiensis pupae and its impact on sterile male yield. Parasites & Vectors 8, 259

Phelps RJ, Burrows PM. 1969 Prediction of the pupal duration of Glossina morsitans orientalis Vanderplank under field conditions. J. Applied Ecol. 6, 323–37.

Saltelli A, Chan R, Scott FM. 2008 Sensitivity analysis. Wiley. 494 p. ISBN: 978-0-470-74382-9.

Saunders DS. 1962 Age determination for female tsetse flies and the age compositions of samples of Glossina pallidipes Aust., G. palpalis fuscipes Newst. and G. brevipalpis Newst. Bull. Entomol. Res. 53, 579-595.

Warren DL, Seifert SN. 2011 Ecological niche modeling in Maxent: the importance of model complexity and the performance of model selection criteria. Ecol Appl. Eco Soc America 21, 335–342.
